# Nickel ferrite nanoparticles doped on hollow carbon microspheres as a novel reusable catalyst for synthesis of *N*-substituted pyrrole derivatives

**DOI:** 10.1038/s41598-023-37817-3

**Published:** 2023-07-05

**Authors:** Setareh Mousavi, Hossein Naeimi, Amir Hossein Ghasemi, Shadan Kermanizadeh

**Affiliations:** Department of Organic Chemistry, Faculty of Chemistry, University of Kashan, Kashan, 87317-51167 Islamic Republic of Iran

**Keywords:** Biochemistry, Chemical biology, Chemistry

## Abstract

Pyrroles are widely spread worldwide because of their critical applications, especially pharmacology. An expedition method for one-pot synthesis of *N*-substituted pyrrole derivatives has been presented by a reaction between 2,5-dimethoxytetrahydrofuran and various primary aromatic amines in the presence of NiFe_2_O_4_ anchored to modified carbon hollow microspheres (NiFe_2_O_4_@MCHMs) as a recoverable reactive catalyst. The Classon-Kass method has been used to synthesize the pyrroles in excellent yields and short reaction times in the same direction with green chemistry rules. This reaction was carried out by employing NiFe_2_O_4_@MCHMs as a catalyst to make a simple procedure with short activation energy in water as an accessible, non-toxic, and biodegradable solvent. This catalyst provides a promising pathway to synthesize *N*-substituted pyrroles several times in a row through the recyclability without remarkable loss of its catalytic activity. The NiFe_2_O_4_@MCHMs nanocatalyst was characterized by applying FT-IR, XRD, FE-SEM, TEM, EDS, BET, TGA, VSM, and elemental mapping techniques. Also, the synthesized *N*-substituted pyrrole derivatives were identified using melting point, FT-IR, and ^1^H NMR analyses.

## Introduction

Nitrogen-containing heterocycles are played an essential role in biological compounds. Most of the drugs approved by the united states food and drug administration (FDA) and presently available on the market include nitrogen-containing heterocyclic moieties^1^. It is predicted that more heterocyclic compounds containing nitrogen will be synthesized and used as drugs in the coming years.

Pyrroles are a group of five-membered heterocycles that contain nitrogen in their structure. These heterocycles are among the most critical nitrogen-containing heterocycles in many natural products and drug compounds. Pyrrole-based materials are also finding increasing usage in materials science^2^. These compounds play a unique role in constructing natural structures and intermediates in many biological reactions^3^. Also, the pyrrole derivatives are current in diverse bioactive pharmaceutical molecules such as; anti-inflammatory^4^, antitumor agents^5^, anti-bacterial^6^, and immunosuppressants^7^. However, the applications of pyrroles are not limited to biological and medicinal compounds. Many derivatives of pyrroles, especially *N*-substituted pyrroles, are widely used in dyes^8^, agrochemicals^9^, polymerization^10^, and solar cells^11^.

Due to the widespread use of pyrroles, various synthetic methods for production of *N*-substituted pyrroles have been developed. *N*-substituted pyrroles can be obtained from a variety of raw materials. One of the most widely used raw materials for preparation of *N*-substituted pyrroles is 2,5-dimethoxy tetrahydrofuran. This method is known as the Klasson-Cass reaction^12^, can be prepared *N*-substituted pyrroles from the reaction between 2,5-dimethoxy tetrahydrofuran and amines. Banik et al., by using bismuth(III) nitrate as a catalyst synthesized the *N*-substituted pyrrole under ultrasonic conditions^13^. Zhang et al. synthesized a wide range of *N*-substituted pyrroles using an antimony magnetic catalyst in water as a solvent^14^. Naeimi et al. prepared the *N*-aryl pyrroles using ionic liquid, [H-NMP][HSO_4_] at room temperature in the green solvent^15^.

The catalysts are essential in many chemical reactions^16–19^. They can reduce the reaction activation energy and time of the reactions^20,21^. One of the main factors that make new catalysts economically attractive is reduced reaction time and increased reaction efficiency^22–25^. Scientists are always looking for ways to improve catalysts^26–29^. Using nanoscience to design new catalysts revolutionized the use of high-performance catalysts^30,31^.

Hollow structures are a group of compounds named based on their texture and structure. Usually, a high percentage of hollow structures is the empty space^32^. One of the standard methods of classifying hollow structures is to organize them based on their shape and form^33^. Also, the hollow structures are classified based on their shell compositions. Hollow structures can exist in single-layer or multi-layer cylindrical, cubic and spherical forms. Hollow spherical structures can be categorized into monolayer spheres, multi-layer spheres^34^, and yolk shells^35^. Hollow spheres are widely used in drug delivery^36–38^, biosensors^39,40^, catalysts^41–44^, and batteries^45^. Many catalytic reactions have been carried out in hollow spherical structures^46^. Spherical hollow structures have a very high contact surface, which has led to their widespread use as a suitable substrate for the placement of nanoparticles. These NPs can be located on the inner and outer surfaces of spheres. This unique property significantly increases the active surface of the catalyst. Increasing the catalyst's active sites boosts the efficiency and reduces the reaction time.

The contact surface area of hollow spheres increases significantly when their walls are porous. Also, optimal use of the effective surfaces of the catalyst substrate increases the active catalytic sites and as a result, increases the reaction efficiency and significantly reduces the reaction time. The reaction between the raw materials on the surface, inside the channels inside the wall, as well as the inner surface of the hollow spheres, react together and produces the *N*-substituted pyrroles. Furthermore, using the substrate, geometric structure and using the appropriate manufacturing method, and stabilizing metals on the surface of the substrate is one of the features of this project, which has made the designed catalyst have a unique recovery capability.

In this research, we hope to design, prepare and identify spherical hollow nanocatalyst for synthesis of *N*-substituted pyrrole derivatives with the highest efficiency and minimum reaction times. Also, it was decided to recover the catalyst and reused several times. The reaction conditions are optimized in various parameters, such as; type of solvent, temperature, catalyst amount and the molar ratio of substrates.

## Experimental

### Materials and apparatus

The reagents and solvents with high purity were purchased from Merck, Fluka, and Aldrich Chemical Companies. The used amine derivatives in chemical reactions were purified by the standard method. The melting points of synthesized organic compounds were determined with a Thermo scientific 9200-point apparatus. Fourier transform infrared (FT-IR) spectra were obtained as potassium bromide pellets with Thermo Nicolet IMPACT-400 FT-IR spectrophotometer in 400–4000 cm^−1^. The Bruker DRX‐400 spectrometer recorded 1-hydrogen nuclear magnetic resonance (^1^H NMR) spectra in CDCl_3_ solvent and the tetramethylsilane as the internal reference. X-ray powder diffraction (XRD) patterns were reported by Philips X’Pert PW 3040 Powder X-ray diffractometer with CuKa radiation, and the patterns were analyzed with X’pert High score plus. The BELSORP-mini II apparatus (Microtrac BEL, Japan) were calculated as nitrogen adsorption–desorption isotherms at 77 K, and the obtained data were measured by the Brunauer–Emmett–Teller (BET) method. The magnetic properties of the NiFe_2_O_4_@MCHMs catalyst were measured at room temperature using a VSM7300 (Meghnatis Daghigh Kavir Co., Kashan, Iran) in a maximum applied field of 15 kOe. The field emission scanning electron microscope (FE-SEM) of the surface, energy dispersive spectroscopy (EDS), and the map scan of modified carbon hollow microspheres were performed on TE-SCAN MIRA3 apparatus that operated at a 15 kV accelerating voltage.

### General procedure for preparation of NiFe_2_O_4_@MHCMs

#### Procedure for preparation of carbon@SiO_2_ spheres

At first, 60 mL of ethanol, 15 mL of deionized water, and 3 mL of aqueous ammonia (25%) were transferred to 250 mL round-bottom flasks equipped with a magnetic stirrer. After a homogeneous mixture was obtained, 2.88 mL of tetraethyl orthosilicate was added dropwise to the reaction mixture over 5 min. The reaction mixture was stirred at room temperature for 30 min with a magnetic stirrer. Then, to the reaction mixture added 1.1 mL of formaldehyde and 0.42 g of resorcinol. The reaction mixture was stirred at room temperature for 18 h. The brown precipitates were collected by the centrifuge at 5 min and 5500 rpm and washed three times with deionized water and ethanol. The wet carbon@SiO_2_ spheres are transferred to the freeze dryer at − 60 °C for 24 h. Finally, for carbonization, the dried carbon@SiO_2_ spheres were heated up to 450 °C for 6 h under pure argon atmosphere.

#### Procedure for preparation of hollow carbon spheres

To remove the SiO_2_ core of carbon@SiO_2_ spheres and prepare hollow carbon spheres, at first 60 mg of carbon@SiO_2_ spheres and hydrofluoric acid solution (20%) were transferred to PTFE (Teflon) beaker equipped with a magnetic stirrer. The mixture was stirred at room temperature for 6 h; finally, the precipitates were collected by centrifuge at 5 min and 5500 rpm, washed with deionized water and ethanol three times, and dried at 80 °C for 12 h.

#### Procedure for preparation of NiFe_2_O_4_@MCHMs

First, 30 mg of hollow carbon spheres were dispersed in 100 mL of deionized water by ultrasound irradiation (30 W, 15 min). Next, a mixture of 0.5 mmol of nickel (II) nitrate and 0.6 mmol of iron (III) nitrate was added to the hollow carbon spheres. After 5 min, 3 mL of aqueous ammonia (25%) was added to the mixture. The mixture was stirred overnight at room temperature**.** Then, it was transferred to a Teflon-lined stainless-steel autoclave for hydrothermal process**,** and placed in an electric oven at 200 °C for 2 h. The precipitates were collected by centrifuge at 5 min and 5500 rpm, washed three times with deionized water and ethanol, and dried at 80 °C for 12 h.

### General procedure for the synthesis of *N*-substituted pyrrole derivatives

For the synthesis of *N-*substituted pyrrole derivatives, a mixture of 2,5-dimethoxy tetrahydrofuran (1 mmol), aniline derivatives (1 mmol), NiFe_2_O_4_@MCHMs (2 mg), and distilled water **(**5 mL**)** were added to 25 mL round-bottom flasks equipped with a heater stirrer and stirred at 50 °C in oil bath. Periodic samples of the reaction were taken and analyzed using thin layer chromatography (TLC). After the reaction was completed and cooled to room temperature, the precipitates were dissolved in chloroform (3 mL). The catalyst was collected by an external magnet, washed with distilled water and acetone, and dried at 80 °C for reuse. A rotary evaporator removed the solvent to obtain the crude product. Finally, the crude product was recrystallized from ethanol to gain pure products.

#### Spectroscopic and physical data:

The synthesized organic compounds were characterized by melting point, FT-IR, and ^1^H NMR analyses. Also, the synthesized compounds were named from **3a** to **3l** as follows.

*1-phenyl-1H-pyrrole* (**3a**); Brown solid; m.p.: 59–62 °C, decompose (Lit. m.p. 60–62 °C)^47^; IR (KBr): *v* = 3033 (C–H, sp^2^ stretch), 1497, 1597 (C=C Ar),746, 692 (C-H, sp^2^ OOP) cm^−1^; ^1^H NMR (400 MHz, CDCl_3_) δ (ppm): 6.37 (d, 2H, *J* = 2.0 Hz), 7.06 (d, 2H, *J* = 2.0 Hz), 7.26–7.41 (m, 5H).

*1-(4-methoxyphenyl)-1H-pyrrole* **(3b)**; Black solid; m.p.: 87–89 °C, decompose (Lit. m.p. 88–89 °C)^48^; IR (KBr): *v* = 3033 (C–H, sp^2^ stretch), 2926 (C–H, sp^3^), 1597 (C=C, Ar), 1310 (C–O), 749 (C–H sp^2^ OOP) cm^−1^; ^1^H NMR (400 MHz, CDCl_3_) δ (ppm): 3.84 (s, 3H), 6.33 (s, 2H), 6.95 (d, 2H, *J* = 8.4 Hz), 7.01 (s, 2H), 7.31(d, 2H, *J* = 8.4 Hz).

*1-(3-methoxyphenyl)-1H-pyrrole* (**3c**); Yellow solid; m.p.: 57–58 °C, decompose (Lit. m.p. 57–58 °C)^49^; IR (KBr): *v* = 3000 (C–H, sp^2^), 2850 (C–H sp^3^), 1400–1600 (C=C, Ar), 1300 (C–O), 743 (C–H sp^2^ OOP) cm^−1^; ^1^H NMR (400 MHz, CDCl_3_) δ (ppm): 3.86 (s, 3H), 6.36 (s, 2H), 6.81 (d, 1H, *J* = 8.0 Hz), 6.95 (s, 1H), 7.01 (d, 1H, *J* = 8.0 Hz), 7.10 (s, 2H), 7.31–7.35 (t, 1H, *J* = 8.4 Hz).

*1-(4-bromophenyl)-1H-pyrrole* (**3d**); Yellow solid; m.p.: 92–94 °C, decompose (Lit. m.p. 93–94 °C)^50^; IR (KBr): *v* = 3130 (C–H, sp^2^), 1497, 1590 (C=C, Ar), 727(C–H sp^2^ OOP), 512(C–Br) cm^−1^; ^1^H NMR (400 MHz, CDCl_3_) δ (ppm): 6.36 (d, 2H, *J* = 2.0 Hz), 7.05 (d, 2H, *J* = 2.0 Hz), 7.26 (d, 2H, *J* = 9.2 Hz), 7.53 (d, 2H, *J* = 8.8 Hz).

*1-(4-chlorophenyl)-1H-pyrrole* (**3e**); Yellow solid; m.p.: 86–88 °C, decompose (Lit. m.p. 86.5–87.5 °C)^50^; IR (KBr): *v* = 3102 (C–H, sp^2^ stretch), 1501 (C=C, Ar), 728 (C–Cl) cm^−1^; ^1^H NMR (400 MHz, CDCl_3_) δ (ppm): 6.36 (d, 2H, *J* = 2.0 Hz), 7.05 (d, 2H, *J* = 1.6 Hz), 7.32 (d, 2H, *J* = 8.8 Hz), 7.38 (d, 2H, *J* = 8.8 Hz).

*1-(3-chlorophenyl)-1H-pyrrole* **(3f.)**; Black solid; m.p.: 51–52 °C, decompose (Lit. m.p. 50.5–51.5 °C)^50^; IR (KBr): *v* = 3102 (C-H, sp^2^ stretch), 1500 (C=C, Ar), 720–820 (C–Cl) cm^−1^; ^1^H NMR (400 MHz, CDCl_3_) δ (ppm): 6.36 (s, 2H), 7.07 (s, 2H), 7.21 (d, 1H *J* = 6.8 Hz), 7.27 (d, 1H, *J* = 8.4 Hz), 7.33–7.37 (t, 1H, *J* = 8.0 Hz), 7.40 (s, 1H).

*1-(2-chlorophenyl)-1H-pyrrole* (**3g**); Black solid; m.p.: 85–86 °C, decompose (Lit. m.p. 86–87 °C)^51^; IR (KBr): *v* = 3050 (C-H, sp^2^ stretch), 1500 (C=C, Ar), 720–820 (C–Cl) cm^−1^; ^1^H NMR (400 MHz, CDCl_3_) δ (ppm): 6.43 (d, 2H, J 4.0 Hz, 2CH), 7.18 (d, 2H, J 4.0 Hz, 2CH), 7.61–7.65 (t, 1H, J 8.0 Hz, CH), 7.74–7.76 (t, 1H, J 4.0 Hz, CH), 8.11 (d, 2H, J 8.0 Hz, 2CH).

*4-(1H-pyrrol-1-yl)phenol* (**3h**); Brown solid; m.p.: 113–116 °C, decompose (Lit. m.p. 113–115 °C)^52^; IR (KBr): *v* = 3448 (O–H, stretch), 3135 (C–H, sp^2^), 1519 (C=C, Ar), 1024 (C–O, stretch), 732 (C–H sp^2^ OOP) cm^−1^; ^1^H NMR (400 MHz, DMSO-*d*_6_) δ (ppm): 6.18 (s, 2H), 6.51 (s, 1H), 6.80 (d, 2H, *J* = 8.8 Hz), 7.16 (s, 2H), 7.31 (d, 2H, *J* = 8.4 Hz).

*1-(p-tolyl)-1H-pyrrole* (**3i**); Black solid; m.p.: 83–84 °C, decompose (Lit. m.p. 84–85 °C)^53^; IR (KBr): *v* = 3050 (C–H, sp^2^ stretch), 2922 (C–H, sp^3^), 1509, 1616 (C=C, Ar) cm^−1^; ^1^H NMR (400 MHz, CDCl_3_) δ (ppm): 2.38 (s, 3H), 6.34 (s, 2H), 7.01 (s, 2H), 7.23 (d, 2H, *J* = 8.0 Hz), 7.27 (d, 2H, *J* = 8.0 Hz).

*1-(naphthalen-1-yl)-1H-pyrrole* (**3j**); Brown solid; m.p.: 119–120 °C, decompose (Lit. m.p. 120 °C)^54^; IR (KBr): *v* = 3054 (C–H, sp^2^ stretch), 1596, 1485 (C=C, Ar), 728 (C–H sp^2^ OOP) cm^−1^; ^1^H NMR (400 MHz, CDCl_3_) δ (ppm): 6.45 (s, 2H, 2CH), 7.04 (s, 2H, 2CH), 7.50–7.56 (q, 4H, J 6.8 Hz, 4CH), 7.79 (d, 1H, J 7.2 Hz, CH), 7.90–7.96 (q, 2H, J 7.6 Hz, 2CH).

*1-(4-nitrophenyl)-1H-pyrrole* (**3k**); Yellow solid; m.p.: 186–187 °C, decompose (Lit. m.p. 185–186 °C)^53^; IR (KBr): *v* = 3150 (C–H, sp^2^), 1616 (C=C, Ar), 1527 (N=O), 1347 (C–N) cm^−1^; ^1^H NMR (400 MHz, CDCl_3_) δ (ppm): 6.44 (s, 2H), 7.19 (s, 2H), 7.52 (d, 2H, *J* = 8.8 Hz), 8.31 (d, 2H, *J* = 9.2 Hz).

*1-(3-nitrophenyl)-1H-pyrrole*, (**3l**); Yellow solid; m.p.: 75–77 °C, decompose (Lit. m.p. 75–76 °C)^50^; IR (KBr): *v* = 3150 (C–H, sp^2^), 1616 (C=C, Ar), 1527 (N=O), 1347 (CN) cm^−1^; ^1^H NMR (400 MHz, CDCl_3_) δ (ppm): 6.42–6.43 (t, 2H, *J* = 2.0 Hz), 7.17–7.18 (t, 2H, *J* = 2.4 Hz), 7.60–7.64 (t, 1H, *J* = 8.4 Hz), 7.73 (d, 1H, *J* = 7.6 Hz), 8.09 (d, 1H, *J* = 8.0 Hz), 8.26–8.27 (t, 1H, *J* = 2.0 Hz).

## Results and discussion

### Preparation and characterization of NiFe_2_O_4_@MCHMs

**Figure 1:**
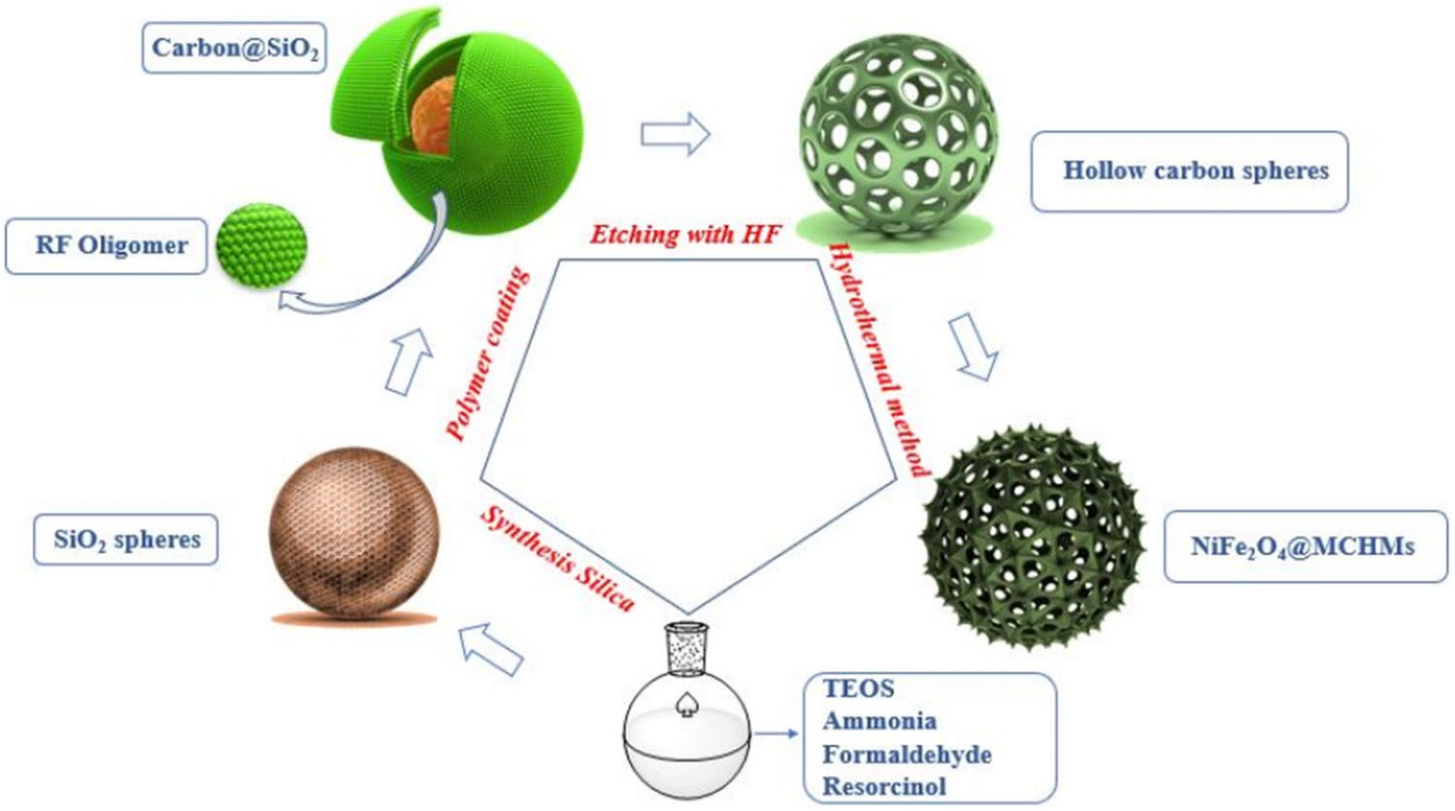
Preparation of NiFe_2_O_4_@MCHMs catalyst.

According to Fig. 1, the preparation of NiFe_2_O_4_@MCHMs catalyst is consisted on the four main steps. In the first step, the SiO_2_ spheres were prepared as hard templates. Next, the SiO_2_ spheres were coated with a resorcinol–formaldehyde (RF) polymer to prepare carbon@SiO_2_ spheres. The wet carbon@SiO_2_ spheres were dried by the freezing method to preserve the porous structure of the resorcinol–formaldehyde (RF) polymer. The carbon@SiO_2_ spheres dried and then were heated to 450 °C under pure argon atmosphere to allow the porous channels on the surface of the spheres to enlarge.

In the next step, the SiO_2_ inside the carbon@SiO_2_ spheres was removed by hydrofluoric acid (HF) to create a hollow structure. Finally, the prepared hollow carbon spheres were functionalized by NiFe_2_O_4_ using the hydrothermal method to enhance the surface and create the effective catalytic capability.

After preparation of the catalyst, various techniques including Fourier-transform infrared spectroscopy (FT-IR), X-ray diffraction analysis (XRD), field emission scanning electron microscopes (FE-SEM), energy dispersive X-Ray analysis (EDX), elemental mapping, Brunauer–Emmett–Teller (BET) theory, vibrating sample magnetometry (VSM), and thermogravimetric analysis (TGA) were used to study and identify the structure, morphology, and functional groups.

**Figure 2:**
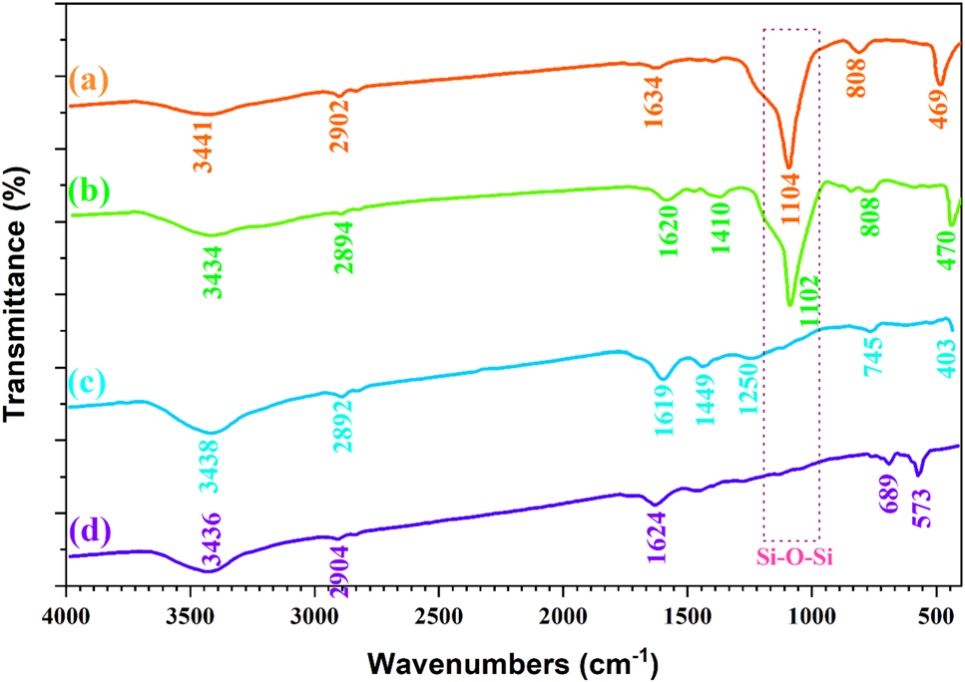
The FT-IR spectra of the carbon@SiO_2_ spheres (**a**), carbon@SiO_2_ spheres after heated to 450 °C under pure argon (**b**), hollow carbon spheres (**c**), and NiFe_2_O_4_@MCHMs catalyst (**d**).

The FT-IR spectra of the carbon@SiO_2_ and carbon@SiO_2_ spheres after being heated to 450 °C under pure argon, hollow carbon spheres, and NiFe_2_O_4_@MCHMs catalyst are shown in Fig. 2.

In FT-IR spectra of the carbon@SiO_2_ spheres (Fig. 2a), the broad peak appeared at 3441 cm^−1^ related to the stretching vibration of O–H bonding of SiO_2_ and the hydroxyl group on resorcinol–formaldehyde (RF) polymer chains. The peak was observed at 2902 cm^−1^ due to the stretching vibration of C–H sp^3^ bonding of the methylene group on RF polymer chains. Finally, the sharp peak that observed at 1104 cm^−1^ corresponds to stretching vibration of the Si–O bonding^55^.

The spectrum of carbon@SiO_2_ spheres after heated to 450 °C under pure argon (Fig. 2b) shows the stretching vibrational peak at 1620 cm^−1^ and 1410 cm^−1^ due to C=C sp^2^ of the aromatics ring for the RF polymer chains. Comparing the two spectra, Fig. 2a,b, it can be seen that after heating the carbon@SiO_2_ under argon gas, no significant change was observed in the RF polymer chains and functional groups. The FT-IR spectrum in Fig. 2c is related to hollow carbon spheres taken after removing the SiO_2_ core. This spectrum lacks peaks around the 1104 cm^−1^ area, which proves the successful removal of the SiO_2_ core and the hollowing the carbon sphere. Finally, the spectrum of the NiFe_2_O_4_@MCHMs catalyst is shown in Fig. 2d. The observed peaks at 689 cm^−1^ and 573 cm^−1^ can be related to the stretching vibration of Fe–O and Ni–O bonding, respectively^56^.

**Figure 3:**
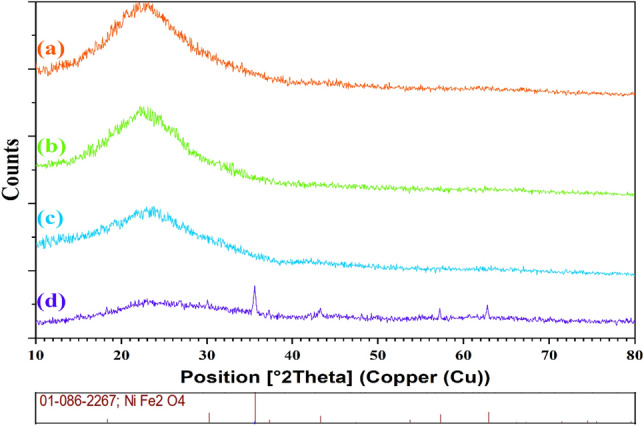
XRD spectra of the carbon@SiO_2_ spheres (**a**), carbon@SiO_2_ spheres after heated to 450 °C under pure argon (**b**), hollow carbon spheres (**c**), and NiFe_2_O_4_@MCHMs catalyst (**d**).

Figure 3 shows the X-ray diffraction (XRD) patterns of the carbon@SiO_2_ spheres (Fig. 3a), carbon@SiO_2_ spheres after heated to 450 °C under pure argon (Fig. 3b), hollow carbon spheres (Fig. 3c), and NiFe_2_O_4_@MCHMs catalyst (Fig. 3d).

The diffraction pattern of the carbon@SiO_2_ spheres (Fig. 3a) shows a broad peak at 2*θ* = 22° attributed to the amorphous structure of the RF polymer on the surface and SiO_2_ in the sphere's core. Additionally, from comparing the carbon@SiO_2_ spheres (Fig. 3a) and carbon@SiO_2_ spheres after heated to 450 °C under pure argon (Fig. 3b), it can be seen that the thermal process has not significantly altered the morphology of the spheres, and only the peak of the amorphous region has become lower. Figure 3c displays the XRD pattern of hollow carbon spheres. The amorphous peak has decreased significantly since the SiO_2_ cores were removed from the spheres in this pattern (Fig. 3c). Finally, after performing the hydrothermal process and modifying the hollow carbon spheres with NiFe_2_O_4_, the morphology of the spheres changed from an amorphous state to a crystalline state. Also, the NiFe_2_O_4_ diffraction pattern contains all the characteristic peaks, which match standard XRD patterns (JCPDS file no. 01-086-2267).

**Figure 4:**
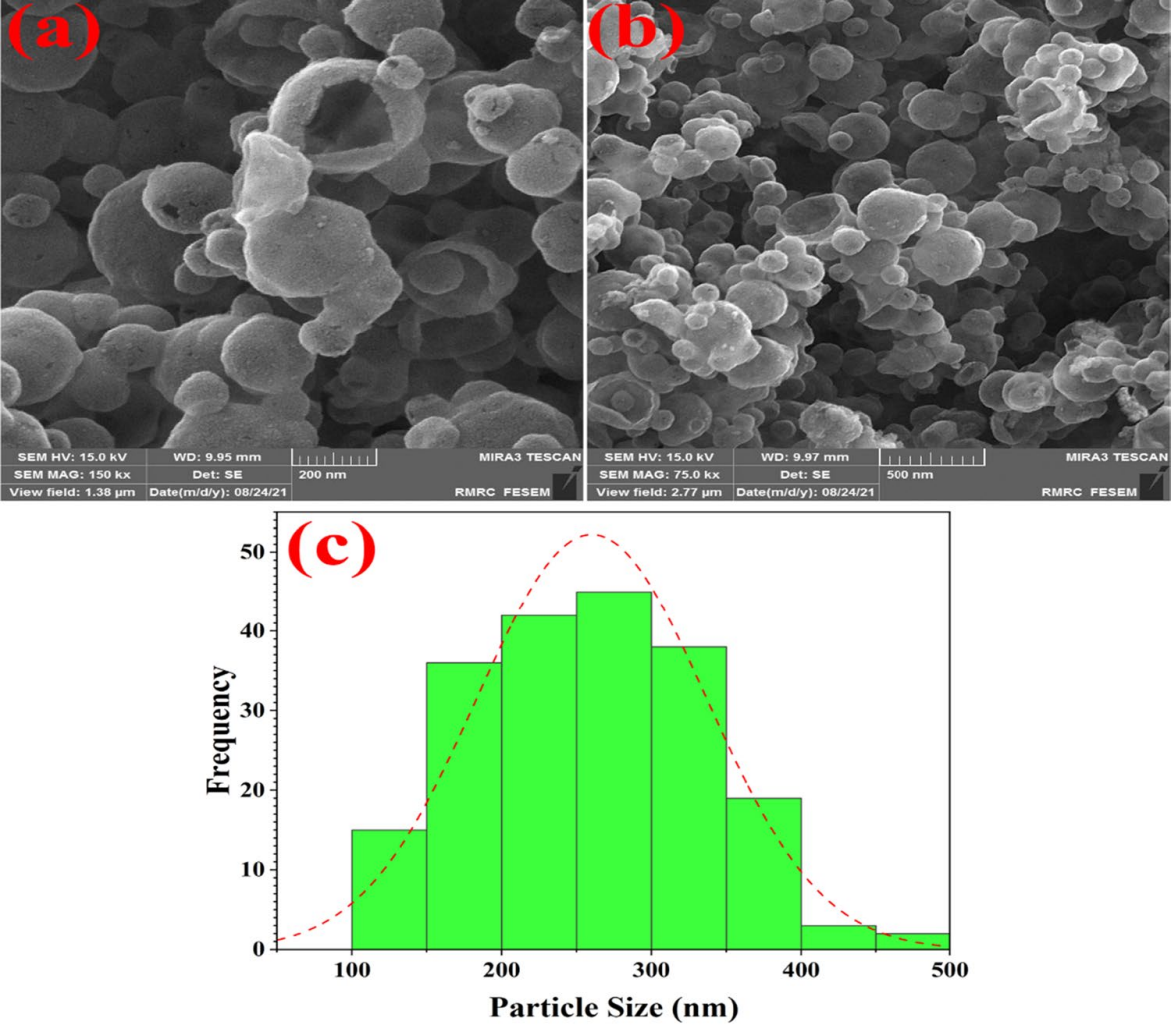
The FE-SEM images of the NiFe_2_O_4_@MCHMs catalyst (**a**,**b**) and the histogram of the size distribution of hollow carbon spheres (**c**).

The NiFe_2_O_4_@MCHMs catalyst's spherical shape is confirmed by the FE-SEM images (Fig. 4a,b). Figure 4a also clearly shows the hollowness of the carbon spheres, which significantly improves the effective surface area of the catalyst. The histogram of the size distribution of hollow carbon spheres is shown in Fig. 4c. The mean size distribution of the hollow carbon spheres was 260.12 nm, with a standard deviation of 76.39 nm obtained. Moreover, the minimum and maximum sizes of the hollow carbon spheres were 104.16 and 483.63 nm, respectively.

**Figure 5:**
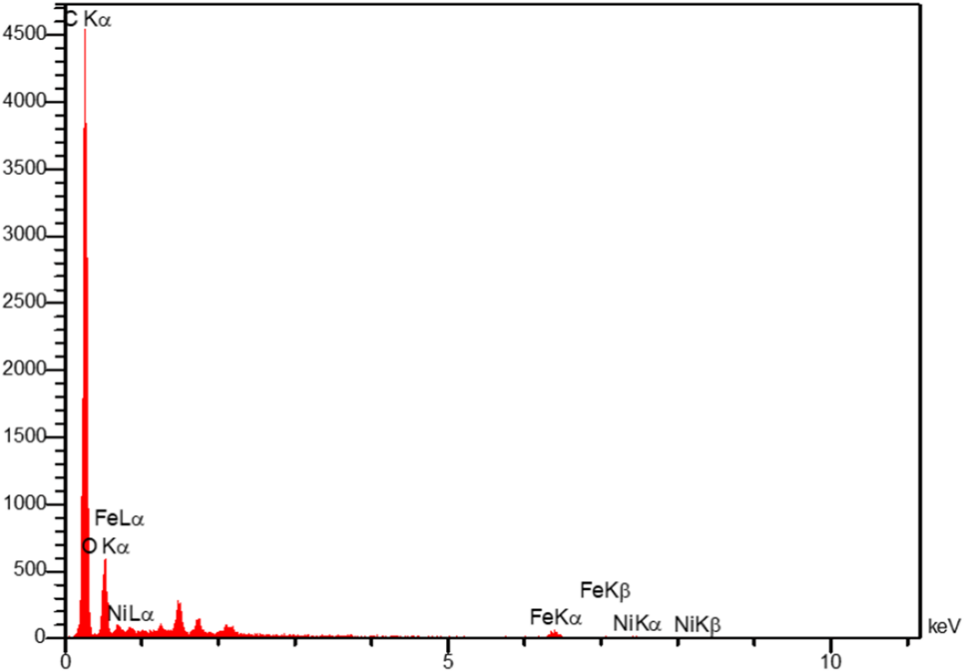
The EDS spectrum of NiFe_2_O_4_@MCHMs catalyst.

Four main elements such as; carbon, oxygen, iron, and nickel were identified by the energy dispersive X-ray analysis of the NiFe_2_O_4_@MCHMs catalyst (Fig. 5). The weight percentages of these elements were calculated as 74.67%, 24.04%, 1.03%, and 0.25%, respectively, which is in reasonable proportion to the used raw materials.

**Figure 6:**
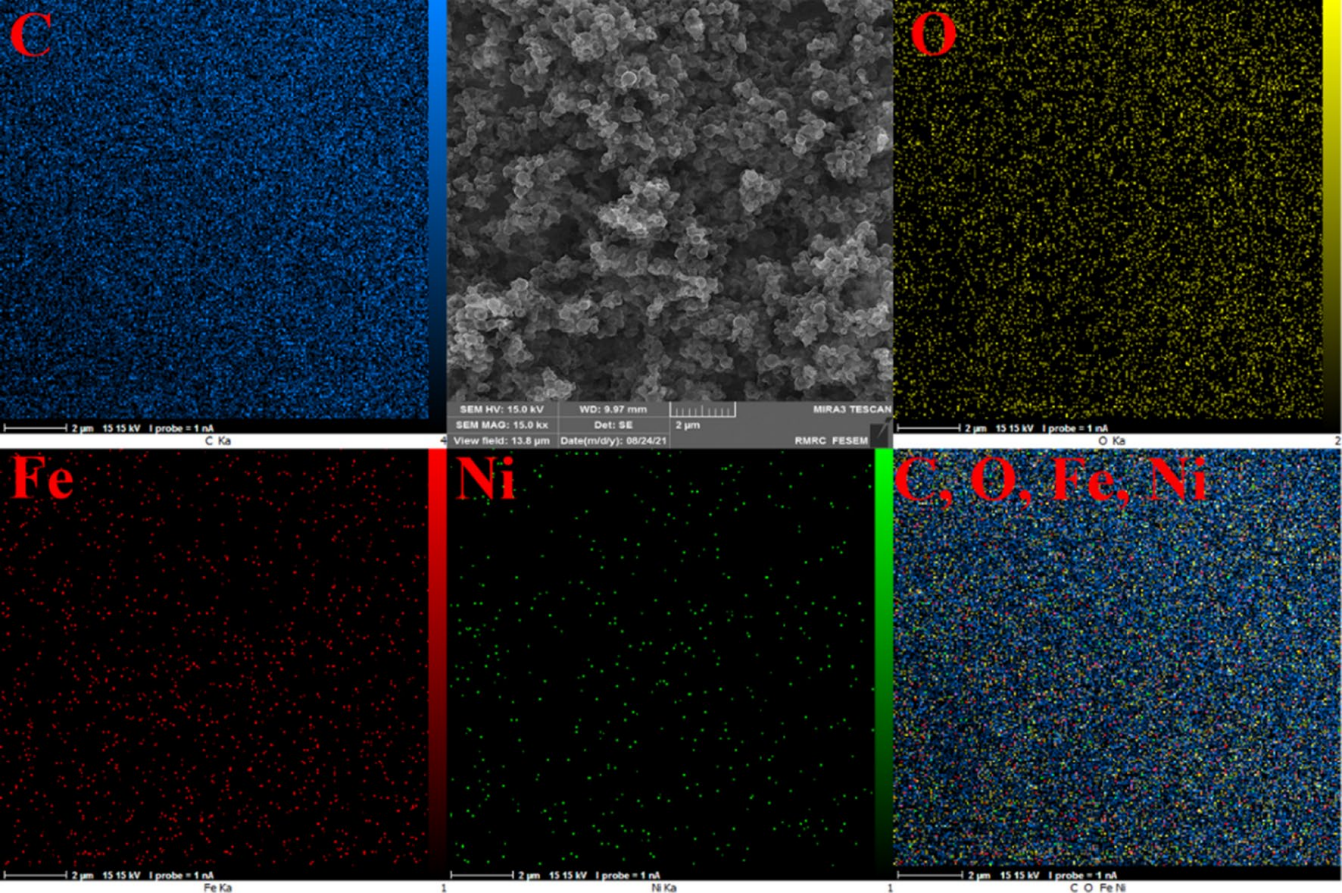
EDX elemental mapping pictures of NiFe_2_O_4_@MCHMs catalyst.

The active surface of the catalyst is increased by the uniform dispersion of nanoparticles on its surface. On the surface of the NiFe_2_O_4_@MCHMs catalyst, uniform dispersion of nanoparticles was found. As shown in Fig. 6, with the proper dispersion of nanoparticles, it was can prove that all surfaces of carbon spheres will exhibit the same catalytic activity.

**Figure 7:**
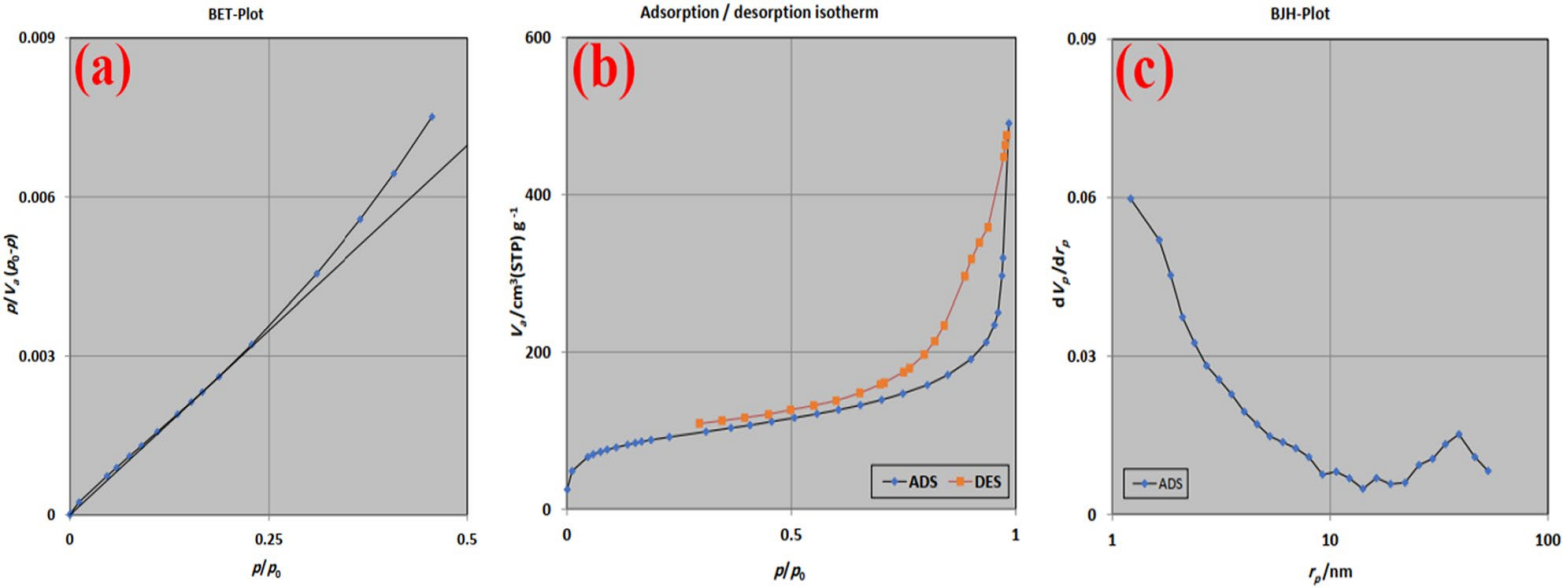
The BET plot (**a**), Adsorption/desorption isotherm (**b**), and BJH plot (**c**) of the NiFe_2_O_4_@MCHMs catalyst.

The BET plot indicates that the NiFe_2_O_4_@MCHMs catalyst sample follows Brunauer–Emmett–Teller theory with a reliable coefficient (Fig. 7a). The surface area of the catalyst was 312.24 m^2^/g measured. A large area of the measured surface is due to the hollow structure of the carbon spheres, which has increased the contact area of the catalyst with the environment.

Also, the catalyst sample's average pore diameter and total pore volume (p/p0 = 0.985) were 9.7241 nm and 0.7591 cm^3^/g, respectively. As a result of the appropriate diameter of the pores on the catalyst's surface, the raw materials can quickly enter the interior of the spheres; therefore, the inner surface of the spheres also functions as an active surface. The mesoporous structure of NiFe_2_O_4_@MCHMs is confirmed by the adsorption/desorption isotherm type of IV (Fig. 7b). Based on the Barrett-Joyner-Halenda (BJH) method, Fig. 7c shows the pore size and pore volume distribution. The total volume of the pores in the synthesized NiFe_2_O_4_@MCHMs catalyst by this method is 0.6378 cm^3^/g.

**Figure 8:**
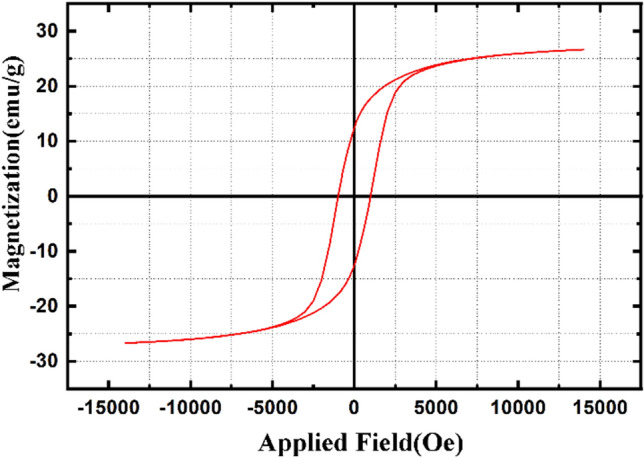
VSM curves for NiFe_2_O_4_@MCHMs catalyst.

The magnetic characteristics of the NiFe_2_O_4_@MCHMs catalyst were investigated using vibrational sample magnetometry (Fig. 8). The magnetization of NiFe_2_O_4_@MCHMs catalyst can be reached the saturation at high fields of 1.5 Tesla. Moreover, the saturation magnetization of the sample is 26.54 emu/g. As a result of this saturation magnetization, the catalyst can easily be collected with the external magnet, allowing easy recovery and reuse.

**Figure 9:**
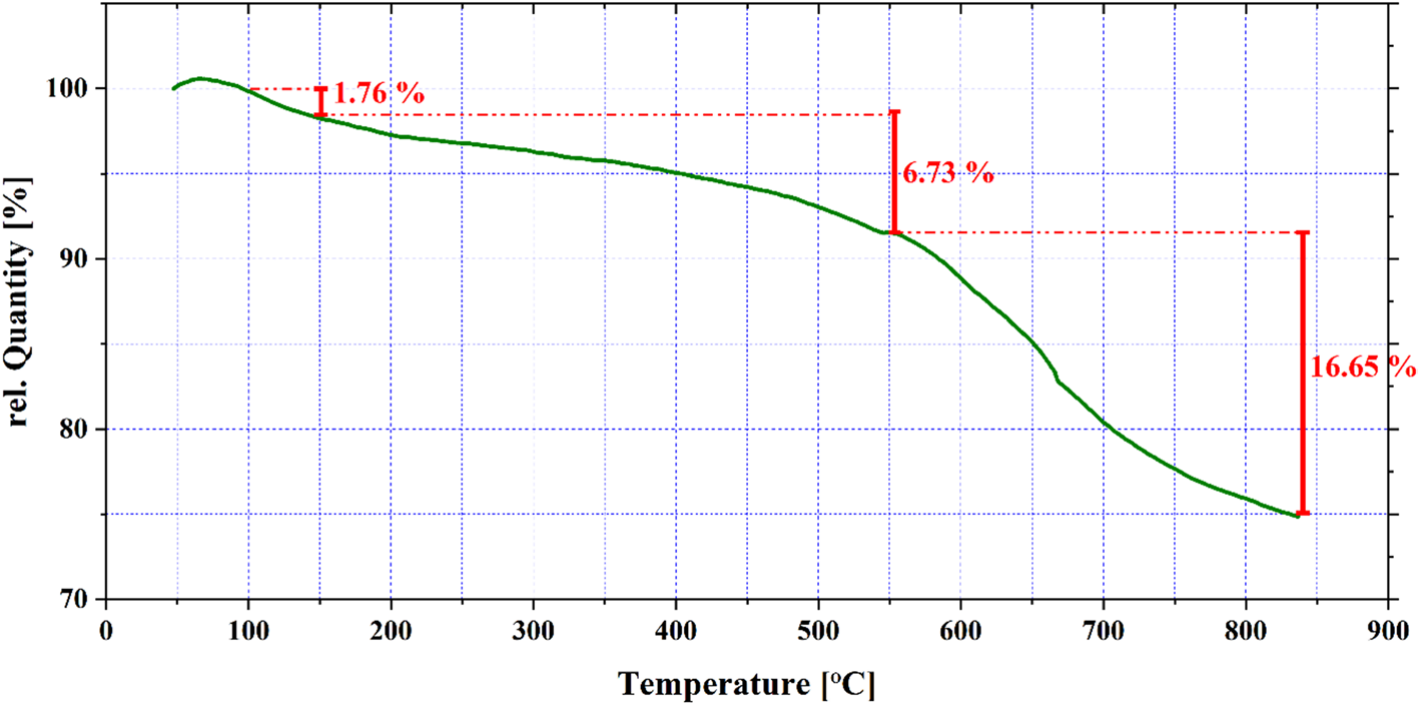
TGA curve of NiFe_2_O_4_@MCHMs catalyst.

Thermogravimetric analysis (TGA) was used to study the thermal stability of the NiFe_2_O_4_@MCHMs, as shown in Fig. 9. The weight loss of roughly 1.76% is detected after heating to 150 °C, which might be attributed to absorbed moisture. Increasing the temperature to 550 °C resulted in a 6.73% weight loss with a moderate slope. As the temperature rises, the weight decreases steadily until it reaches 16.65%, which might be due to resorcinol–formaldehyde (RF) polymer disintegration^57^. The charred mass received at the end of the heating cycle is about 25.14% of the original NiFe_2_O_4_@MCHMs catalyst weight collected.

### Investigation of catalytic activity

To obtain the optimal reaction conditions and use them in the synthesis of *N*-substituted pyrrole derivatives, the reaction between aniline and 2,5-dimethoxy tetrahydrofuran was chosen as a model reaction. The parameters such as; type of solvent, temperature, amount of catalyst, and the molar ratio between aniline and 2,5-dimethoxy tetrahydrofuran were investigated, and the related results are shown in Table 1.

**Table 1:** Optimization of reaction conditions for synthesis of N-substituted pyrrole by NiFe2O4@MCHMs catalyst.


Entry	Catalyst amount (mg)	Solvent	Temp. (°C)	Molar ratio (DMTHF: aniline)	Time (min)	Yield^b^ (%)
1	2	EtOH	Reflux	(1:1)	16	91
2	2	CH_3_CN	Reflux	(1:1)	26	55
3	2	H_2_O	Reflux	(1:1)	4	97
4	2	H_2_O	70	(1:1)	4	97
5	2	H_2_O	50	(1:1)	4	97
6	2	H_2_O	50	(1.2:1)	8	95
7	2	H_2_O	50	(1.4:1)	12	93
8	2	H_2_O	25	(1:1)	42	78
9	2	H_2_O/EtOH (1:1)	Reflux	(1:1)	66	68
10	2	H_2_O/EtOH (1:2)	Reflux	(1:1)	74	50
11	2	DMF	100	(1:1)	48	30
12	4	EtOH	Reflux	(1:1)	18	79
13	4	CH_3_CN	Reflux	(1:1)	34	54
14	4	H_2_O	Reflux	(1:1)	8	95
15	4	H_2_O/EtOH (1:1)	Reflux	(1:1)	78	72
16	4	H_2_O/EtOH (1:2)	Reflux	(1:1)	84	46
17	4	DMF	100	(1:1)	42	39
18	6	EtOH	Reflux	(1:1)	14	77
19	6	CH_3_CN	Reflux	(1:1)	38	51
20	6	H_2_O	Reflux	(1:1)	12	88
21	6	H_2_O/EtOH (1:1)	Reflux	(1:1)	88	44
22	6	H_2_O/EtOH (1:2)	Reflux	(1:1)	98	39
23	6	DMF	100	(1:1)	58	24

^a^Reaction conditions: 2,5-dimethoxy tetrahydrofuran, aniline, NiFe_2_O_4_@MCHMs, and solvent (5 mL).

^b^Isolated yield.

According to the results of Table 1, the highest efficiency has been observed in entries 4 and 5. Considering that in entry 5, the reaction with high efficiency was carried out at a lower temperature than the entry 4, with water as a solvent, a temperature of 50 °C, a 1:1 ratio of raw materials, and 2 mg of NiFe_2_O_4_@MCHMs catalyst as optimal conditions for the reaction model are selected. Also, the reaction efficiency decreased as catalyst amounts increased, which can be attributed to increased connections between the catalyst and raw materials.

**Figure 10:**
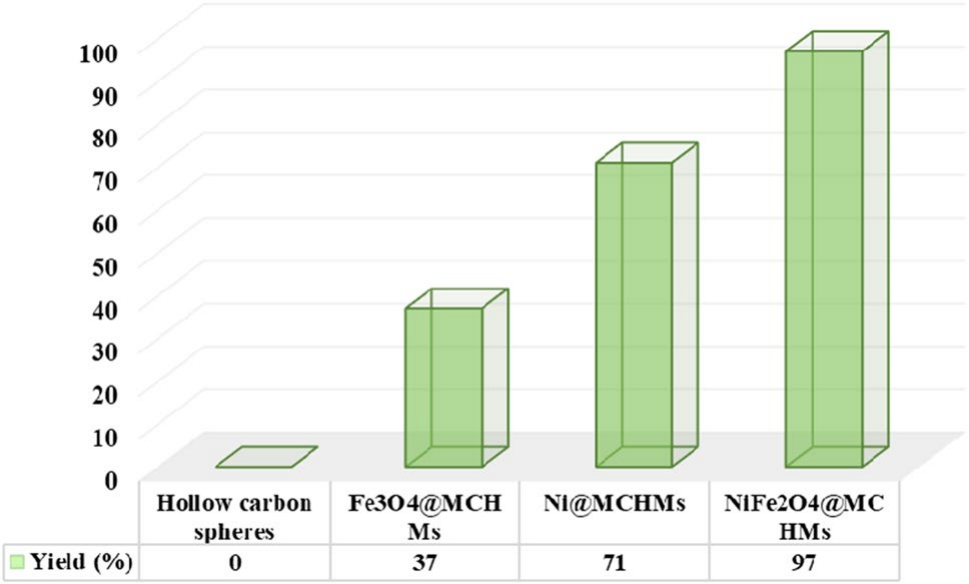
Comparison of catalytic effect between Hollow carbon spheres, Fe_3_O_4_@MCHMs, Ni@MCHMs, and NiFe_2_O_4_@MCHMs for the synthesis of 3a.

To investigate the synergistic effect of the catalyst and its confirmation, the hollow carbon spheres, Fe_3_O_4_@MCHMs, Ni@MCHMs, and NiFe_2_O_4_@MCHMs were prepared. Then, the reaction between aniline and 2,5-dimethoxy tetrahydrofuran was chosen as a model reaction and the product yield was calculated in the presence of each of the prepared catalysts and the related results are shown in Fig. 10. According to the obtained results, the hollow carbon spheres that are used as a catalyst, the reaction has not shown any catalytic effect. It has been observed that the nanocatalysts comprising of NiFe_2_O_4_@MCHMs demonstrate superior efficacy in comparison to those containing Fe_3_O_4_@MCHMs. Finally, the bimetallic sample has shown a much higher efficiency by taking advantage of the synergistic effect.

The reaction was performed in the presence of aniline derivatives containing electron-donating and electron-withdrawing groups after optimizing the solvent, temperature, and catalyst amount.

Table 2 shows the yield and reaction time for each derivative prepared using NiFe_2_O_4_@MCHMs catalyst. All of the *N-*substituted pyrrole derivatives prepared from aniline derivatives with electron-donating and electron-withdrawing groups had high yields and short reaction times. According to the Table 2, anilines with high steric crowding had lower yields than others. Also, higher efficiency and shorter reaction times were observed for aniline derivatives with electron-donating substituents.

**Table 2:**
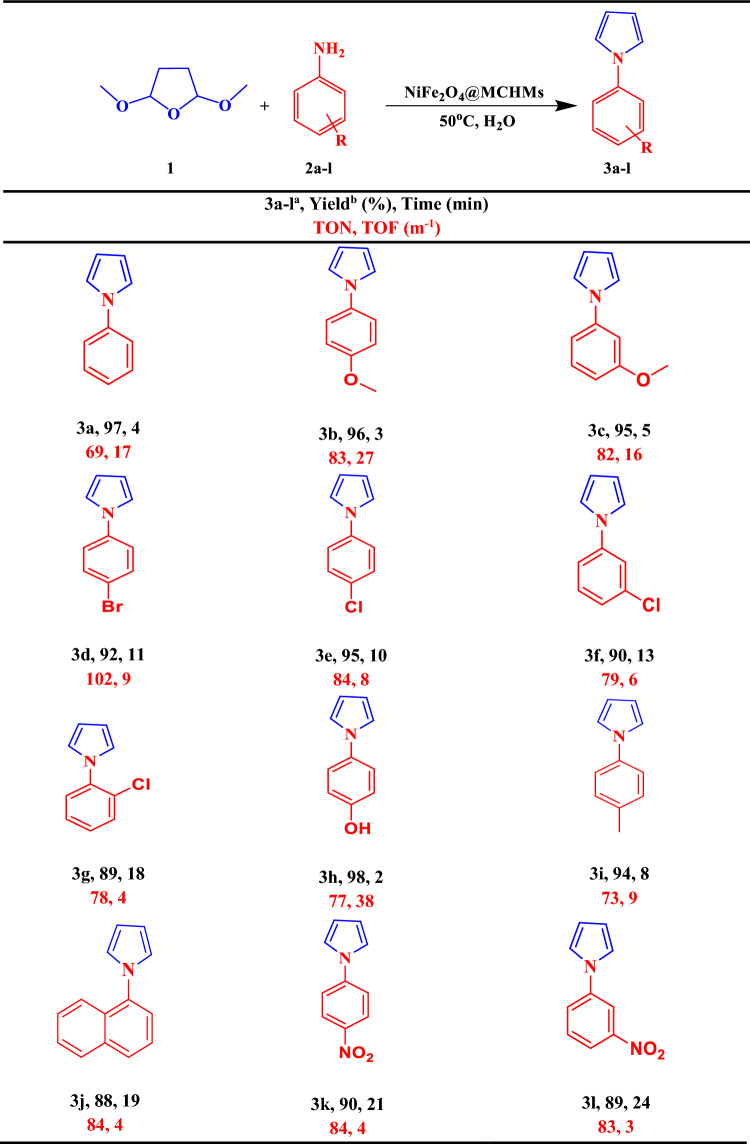
Synthesis of *N*-substituted pyrrole derivatives by NiFe_2_O_4_@MCHMs catalyst.

^a^Reaction conditions: 2,5-dimethoxy tetrahydrofuran (1 mmol), aniline derivatives (1 mmol), NiFe_2_O_4_@MCHMs (2 mg), and H_2_O (5 mL) at 50 °C.

^b^Isolated yield.

The efficiency of NiFe_2_O_4_@MCHMs catalyst was investigated with the previously reported catalysts for the synthesis of 1-phenyl-1H-pyrrole (**3a**) from 2,5-dimethoxy tetrahydrofuran and aniline, and the results are shown in Table 3. The results in this Table show the excellent efficiency of NiFe_2_O_4_@MCHMs catalyst compared to the reported catalysts. In comparison with of NiFe_2_O_4_@MCHMs catalyst, the investigated reported catalysts have lower efficiency in thermal conditions and the required more time to perform the reaction (Table 3, entries 1–4).

**Table 3:** Comparison of the catalytic activity of the NiFe_2_O_4_@MCHMs catalyst with the other reported catalysts for the synthesis of 1-phenyl-1H-pyrrole (3a).

Entry	Catalyst (conditions)	Time (min)	Yield^a^ (%)	References
1	γ-Fe_2_O_3(at)_SiO_2_-Sb-IL (0.08 g, H_2_O, 100 °C)	40	95	^14^
2	Squaric acid (0.5 mol %, H_2_O, 60 °C)	180	95	^58^
3	Sc (OTf)_3_ (3 mol %, 1,4-dioxane, 100 °C)	40	91	^59^
4	MWCNTs–SO_3_H (0.04 g, H_2_O, 80 °C)	45	88	^60^
5	Manganese (II) nitrate tetrahydrate (0.1 mmol, neat, 120 °C, Microwave irradiation)	20	89	^61^
6	Montmorillonite K-10 (0.5 g, ether, Microwave irradiation)	4	90	^62^
**7**	**NiFe**_**2**_**O**_**4**_**@MCHMs (0.002 g, H**_**2**_**O, 50 °C)**	**4**	**97**	**This work**

Significant values are given in bold.

^a^Isolated yield.

Also, entries 5 and 6 of the reaction under microwave irradiation have been investigated, and the results for the present catalyst has higher efficiency than their efficiencies. The reaction efficiency of the NiFe_2_O_4_@MCHMs in thermal conditions is higher than those reported catalysts under microwave irradiation.

### Proposed reaction mechanism

**Figure 11:**
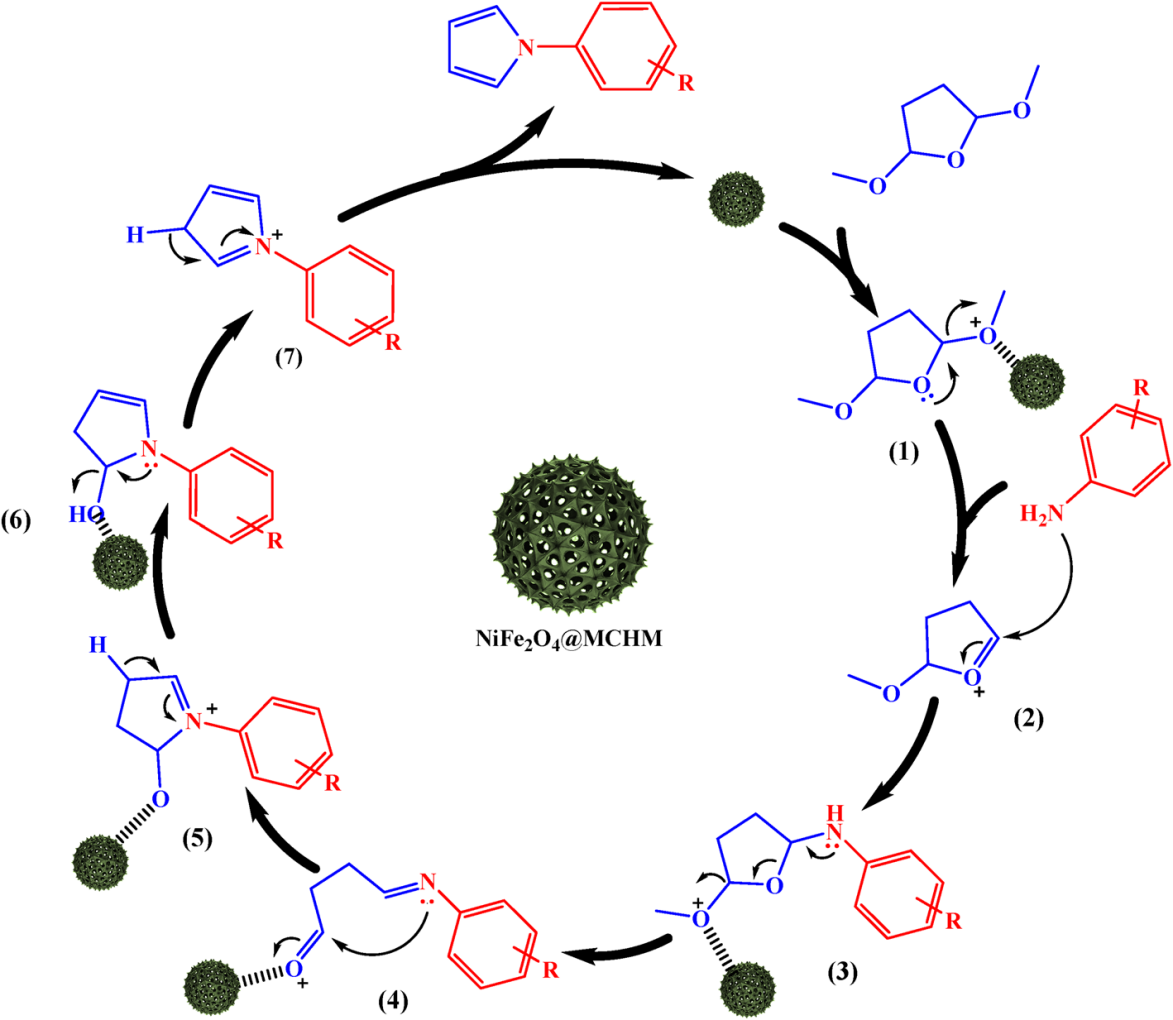
The proposed mechanism for the synthesis of *N*-substituted pyrrole derivatives.

Figure 11 illustrates the proposed reaction mechanism to synthesize *N-*substituted pyrrole derivatives using NiFe_2_O_4_@MCHMs. In the first step, the NiFe_2_O_4_@MCHMs catalyst chelates the methoxy group of the 2,5-dimethoxy tetrahydrofuran **1**, and the non-bonding electron pair of the oxygen in the ring causes the methoxy group to leave. Then, aniline attacks the carbon adjacent to the positively charged oxygen of compound **2**. In the next step, the non-bonding electron pair of nitrogen on compound **3** causes the removal of the methoxy group by forming the imine. In this step, the catalyst has helped remove the methoxy group. Then, the catalyst chelates with the carbonyl group of compound **4** and facilitates the attack of nitrogen on carbonyl and ring closure. The positively charged nitrogen of compound **5** has been neutralized by losing one hydrogen. Next, the hydroxy group of compound **6** chelates with the catalyst and the nitrogen group helped to remove the hydroxy group with its non-bonded electron. Finally, compound **7** has become a product by losing one hydrogen, and the catalyst has entered the reaction cycle again.

### Reusability

**Figure 12:**
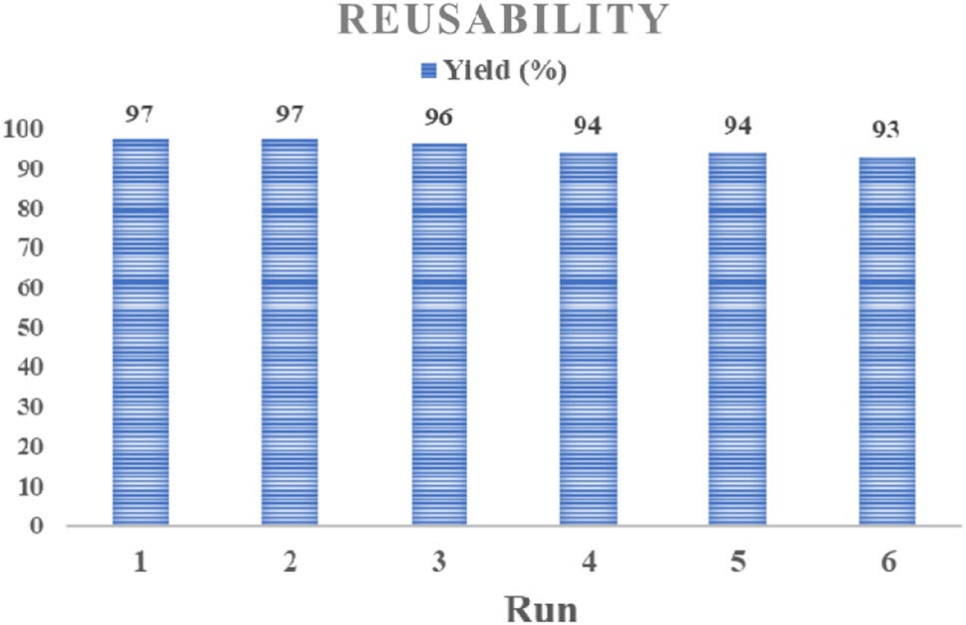
Reusability of the NiFe_2_O_4_@MCHMs catalyst for the synthesis of *N*-substituted pyrrole derivatives.

Considering the importance of recovery and reusability of catalysts, the prepared catalyst was recovered and reused for six runs, and its results are shown in Fig. 12. During the six runs of reusing the catalyst in the model reaction, the yield was minimally reduced, and the performance was excellent.

**Figure 13:**
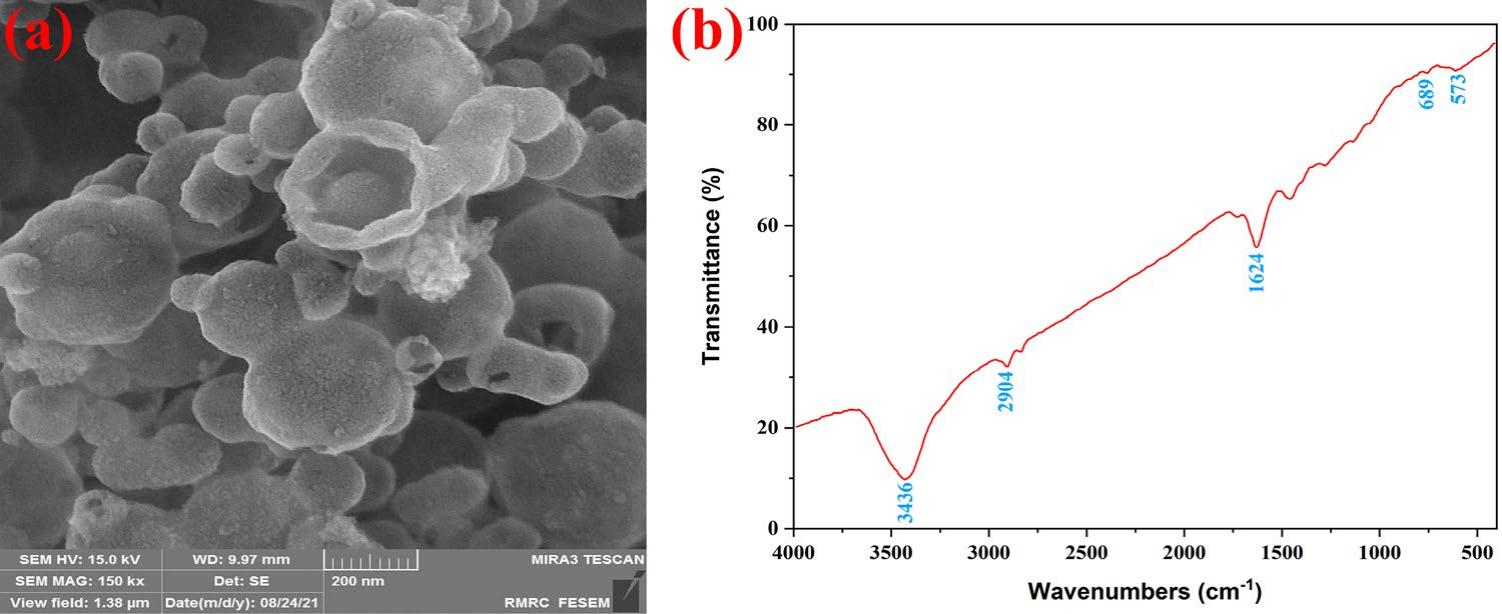
The FE-SEM image (**a**), and the FT-IR spectra (**b**) of the NiFe_2_O_4_@MCHMs catalyst after six runs.

Figure 13a shows the FE-SEM image of the catalyst after six runs. No change has been observed in the morphology of the catalyst after six runs of recovery and reuse, indicating the nanoparticles' strength and stability. The FT-IR spectrum for catalyst (Fig. 13b) was provided after undergoing six cycles of reuse the recovered catalyst. The spectrum of the recovered catalyst was not different from the original sample, so it can be proved that the catalyst remains unchanged in the molecular structure after six cycles of recovery.

An evaluation of inductively coupled plasma atomic emission spectroscopy (ICP-OES) was carried out on the catalyst both prior to and subsequent to its recovery. According to the findings, the NiFe_2_O_4_@MCHMs catalyst contained 0.39 × 10^–4^ mol g^−1^ of Ni and 1.43 × 10^–4^ mol g^−1^ of Fe. In accordance with the analysis of two samples, it appears that the catalyst has maintained its stability even after undergoing six recoveries, as no significant alterations were detected.

Furthermore, the hot filtration method was used to study the leaching of the NiFe_2_O_4_@MCHMs catalyst. An external magnet was used to separate the nanocatalyst from the reaction mixture after 2 min. The reaction progress was monitored using thin layer chromatography (TLC) after heating the filtrate mixture. The monitored reaction did not progress after filtration, so the hot filtration analysis shows the leaching for NiFe_2_O_4_@MCHMs catalyst does not happen.

## Conclusion

In this research, the synthesis of *N*-substituted pyrrole derivatives has been investigated using new hollow nanocatalysts. Among the various methods for preparing *N*-substituted pyrroles, the article's authors have investigated one of the most efficient methods, i.e., the synthesis of *N*-substituted pyrrole derivatives from the reaction of the 2,5-dimethoxy tetrahydrofuran with various aniline derivatives. The prepared catalyst increased the reaction yield and also decreased the reaction times. In addition, the catalyst is easily separated from the reaction mixture and has displayed the ability to be reused with a minor decrease in efficiency. This hollow catalyst and its unique structure has increased the effective contact surface, which makes a smaller amount of catalyst needed for the reaction to progress. All the obtained products were identified using ^1^H NMR and have the highest degree of purity (Supplementary Information S1).

## Supplementary Information

Supplementary Figures.

## Data Availability

Electronic supplementary material contains ^1^H NMR, FT-IR and microscopy data.
