# Supplementary material for: Nickel ferrite nanoparticles doped on hollow carbon microspheres as a novel reusable catalyst for synthesis of N-substituted pyrrole derivatives
Source: Sci Rep. 2023 Jul 5;13:10840. doi: 10.1038/s41598-023-37817-3 (PMC10322829; doi:10.1038/s41598-023-37817-3)
Supplement: Supplementary file 1 — Supplementary Figures. [file 41598_2023_37817_MOESM1_ESM.pdf]

Supplementary Information

Nickel ferrite nanoparticles doped on hollow carbon microspheres as a novel reusable catalyst for synthesis of N-substituted pyrrole derivatives

Setareh Mousavi, Amir Hossein Ghasemi, Shadan Kermanizadeh, Hossein Naeimi*

Department of Organic Chemistry, Faculty of Chemistry, University of Kashan, Kashan, 87317-51167, I.R. Iran; Tel: 98-31-55912388; Fax: 983155912397; E-mail: Naeimi@kashanu.ac.ir

General procedure for the synthesis of *N*-substituted pyrrole derivatives

For the synthesis of *N*-substituted pyrrole derivatives, a mixture of 2,5-dimethoxy tetrahydrofuran (1 mmol), aniline derivatives (1 mmol), $NiFe_2O_4$@MCHMs (2 mg), and distilled water **(**5 mL**)** were added to 25 ml round-bottom flasks equipped with a heater stirrer and stirred at 50 °C in oil bath. Periodic samples of the reaction were taken and analysed using Thin-layer chromatography (TLC). After the reaction was completed and cooled to room temperature, the precipitates were dissolved in chloroform (3 ml). The catalyst was collected by an external magnet, washed with distilled water and acetone, and dried at 80 ° C for reuse. A rotary evaporator removed the solvent to obtain the crude product. Finally, the crude product was recrystallized in ethanol to gain pure products. The synthesized organic compounds were characterized by melting point, FT-IR, and ^{1}H NMR methods.

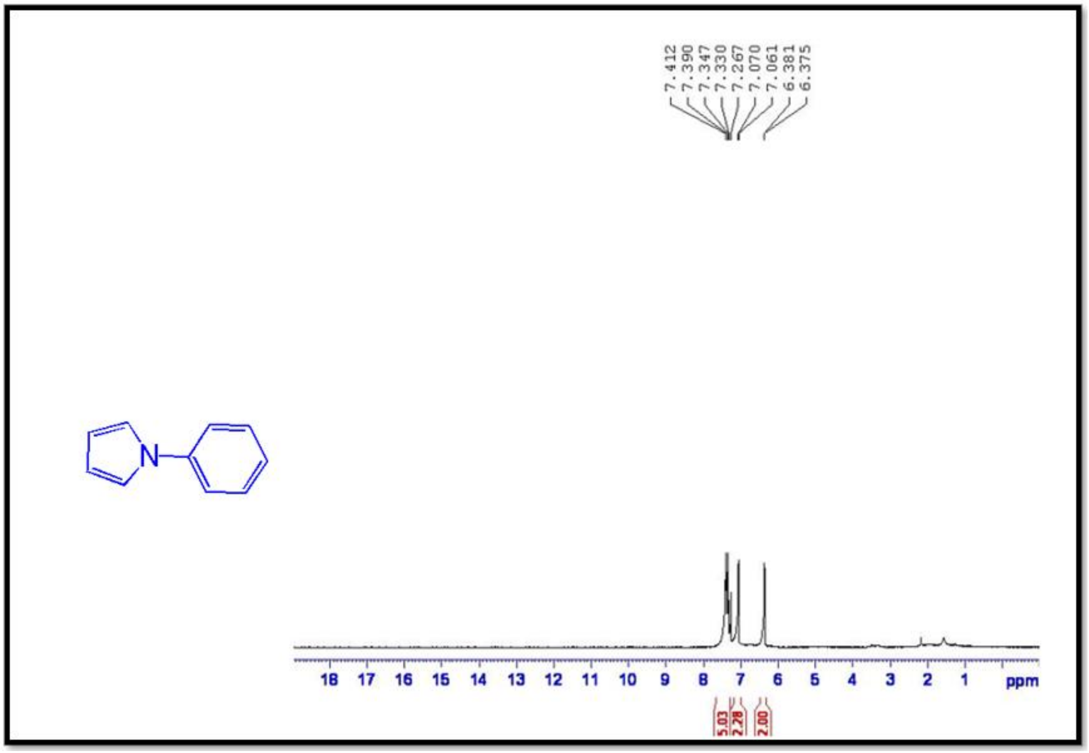

Figure S1. ^{1}H NMR spectra of 1-Phenyl-1H-pyrrole (3a)

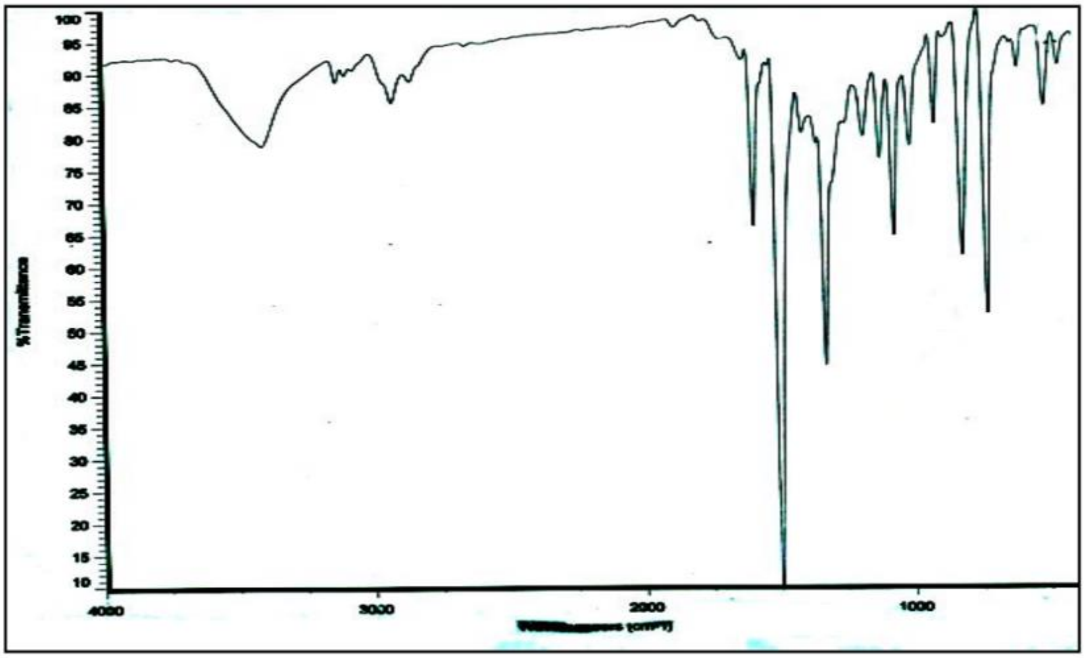

Figure S2. FT-IR of 1-Phenyl-1H-pyrrole (3a)

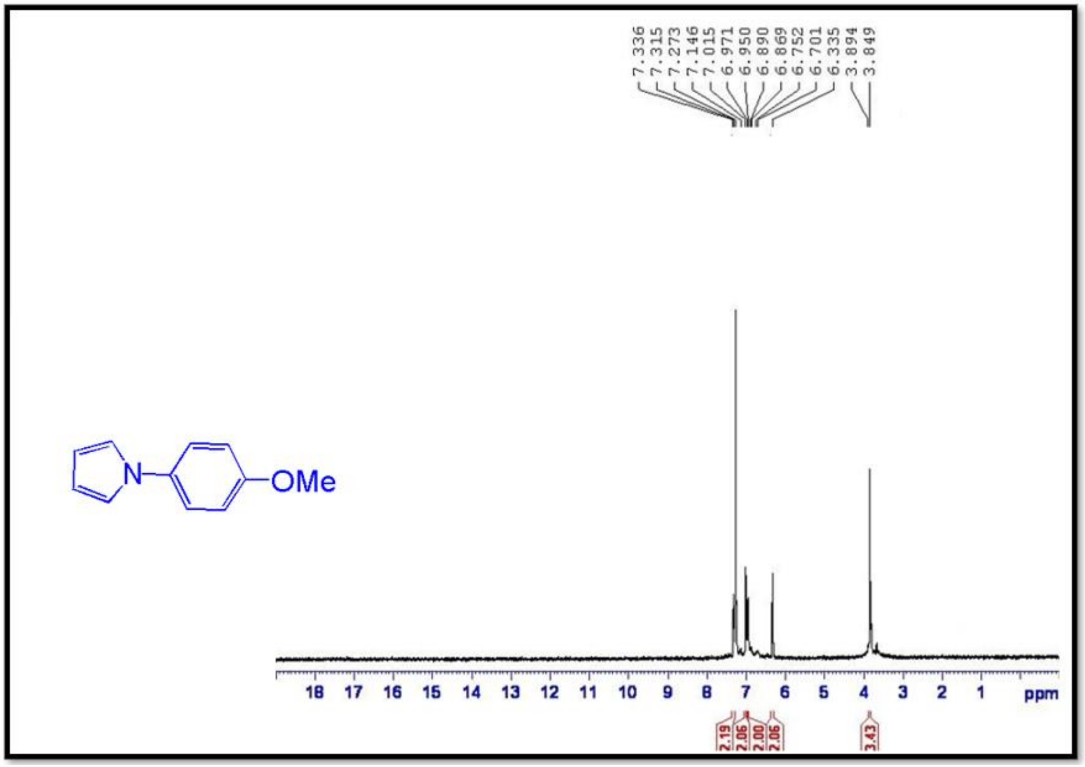

Figure S3. ^{1}H NMR spectra of 1-(4-Methoxyphenyl)-1H-pyrrole (3b)

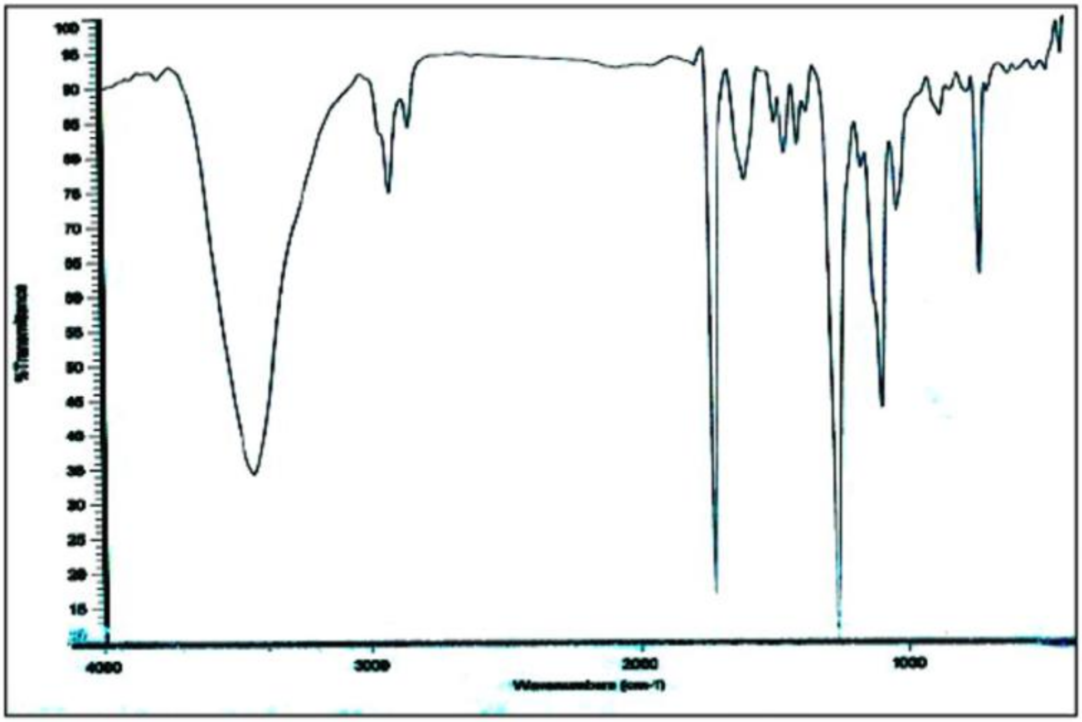

Figure S4. FT-IR of 1-(4-Methoxyphenyl)-1H-pyrrole (3b)

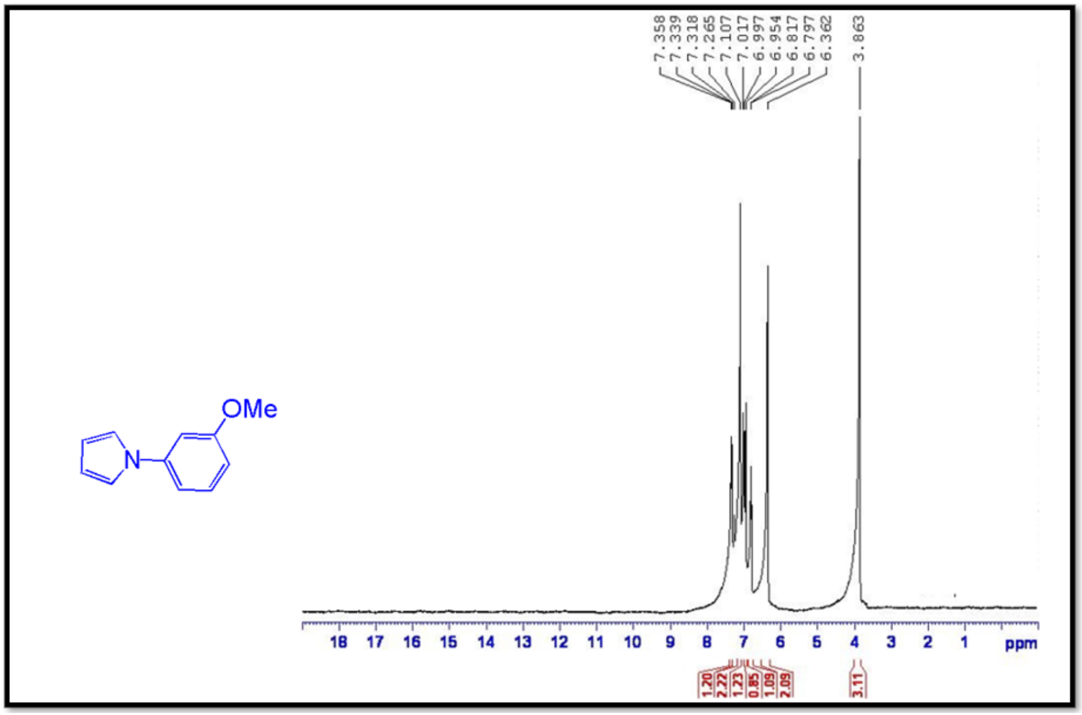

Figure S5. ^{1}H NMR spectra of 1-(3-Methoxyphenyl)-1H-pyrrole (3c)

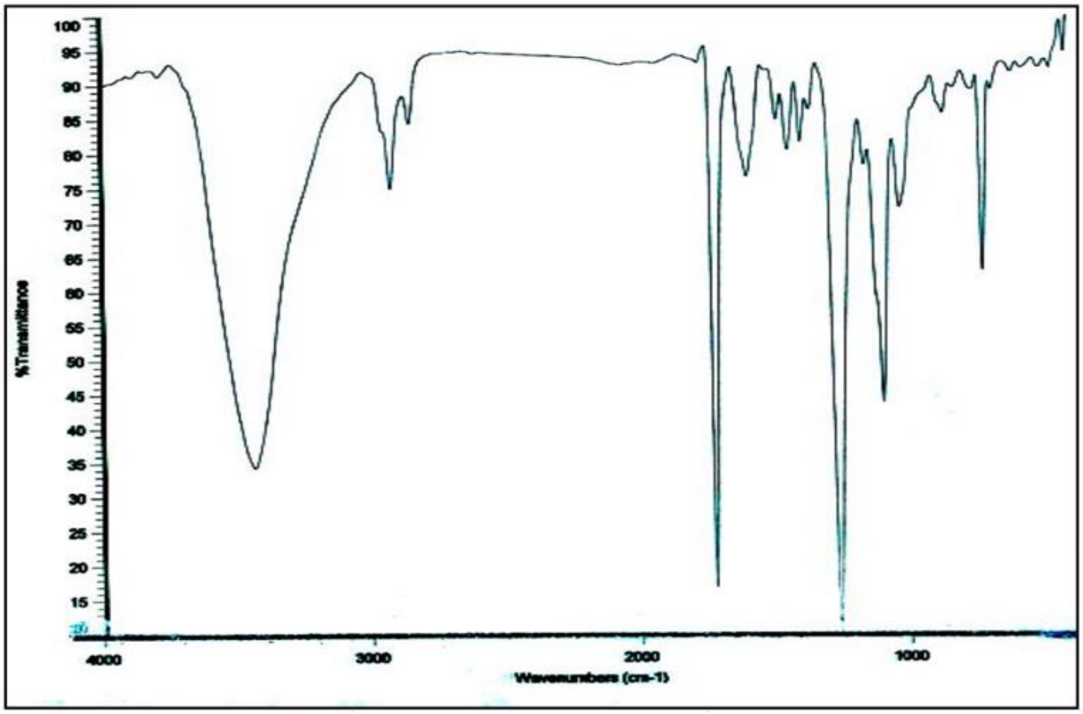

Figure S6. FT-IR of 1-(3-Methoxyphenyl)-1H-pyrrole (3c)

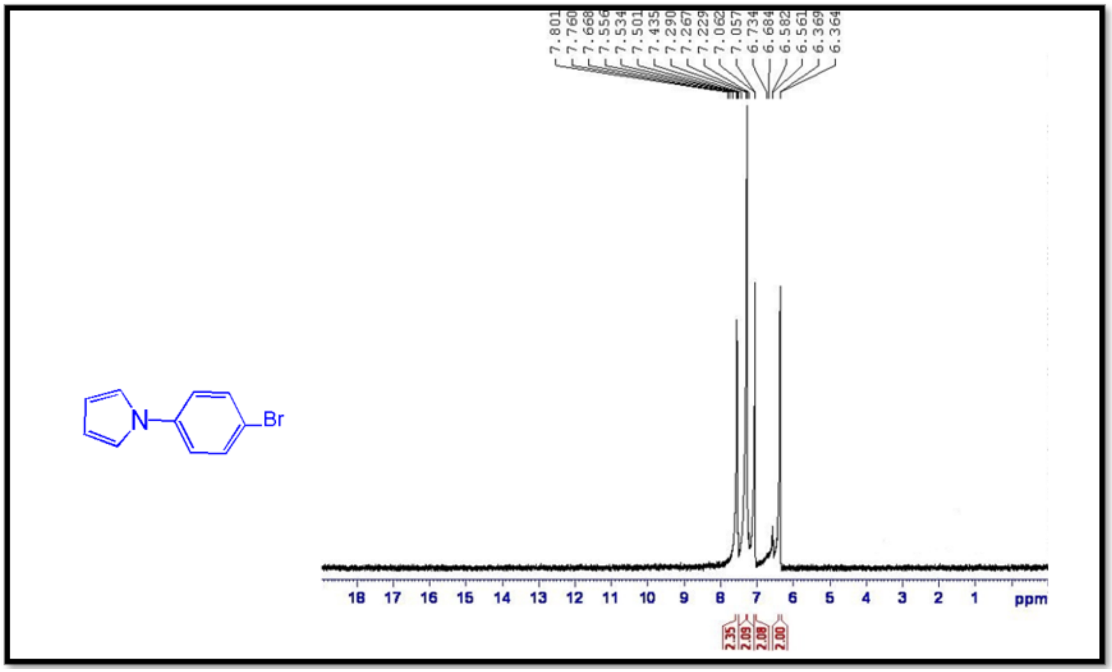

Figure S7. ^{1}H NMR spectra of 1-(4-Bromophenyl)-1H-pyrrole (3d)

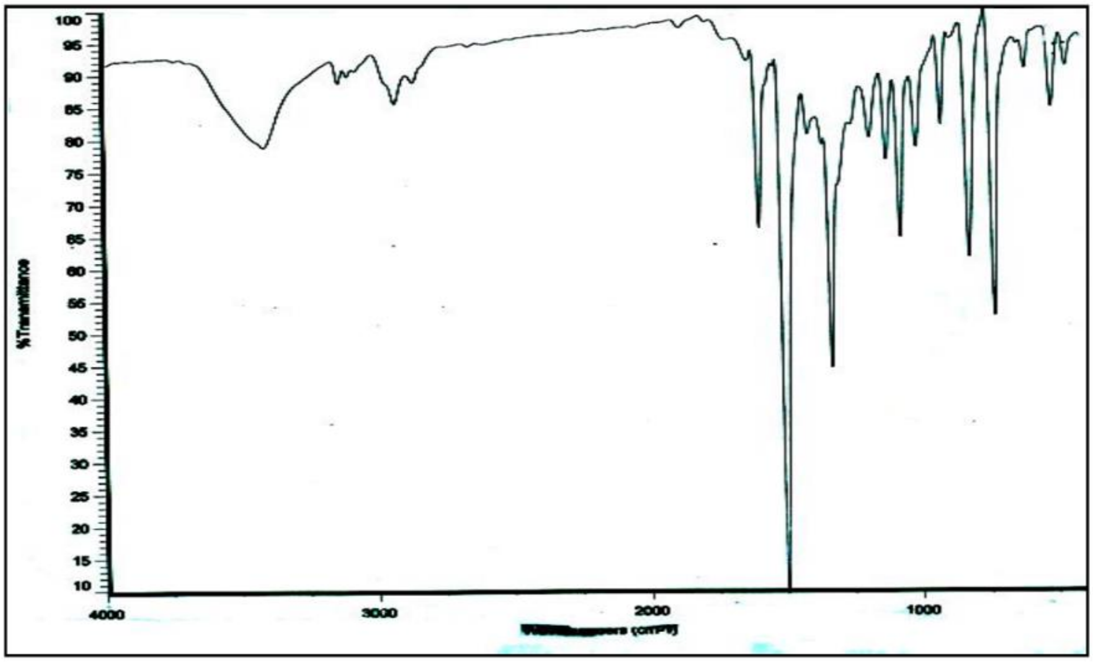

Figure S8. FT-IR of 1-(4-Bromophenyl)-1H-pyrrole (3d)

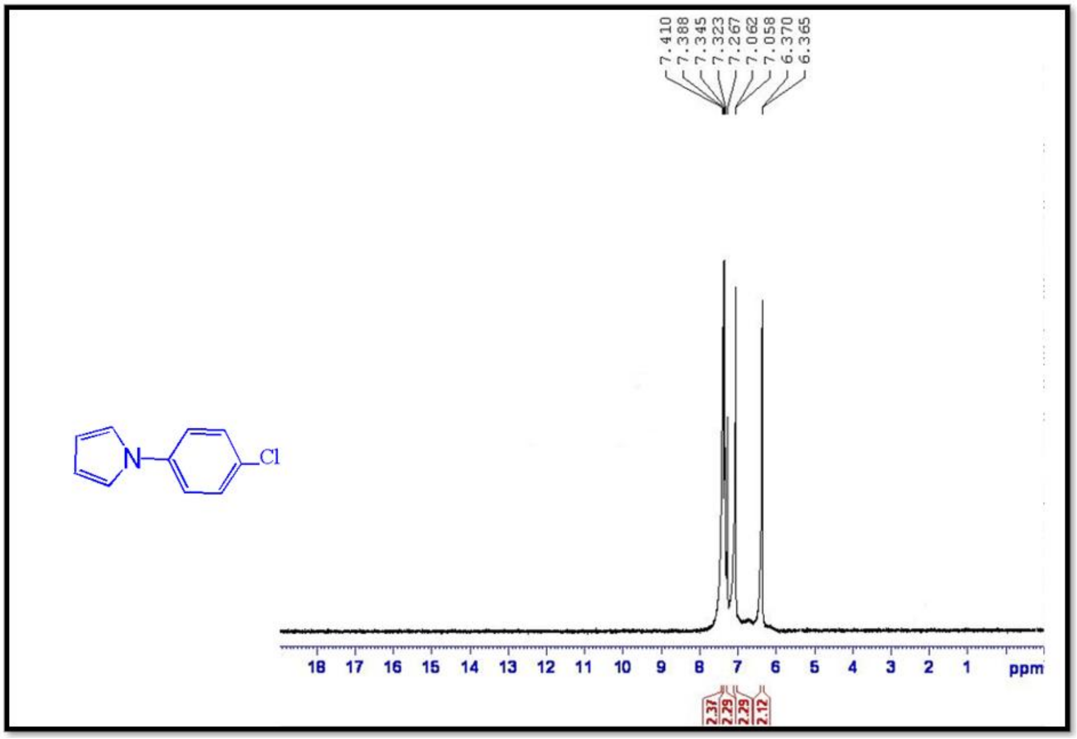

Figure S9. ^{1}H NMR spectra of 1-(4-Chlorophenyl)-1H-pyrrole (3e)

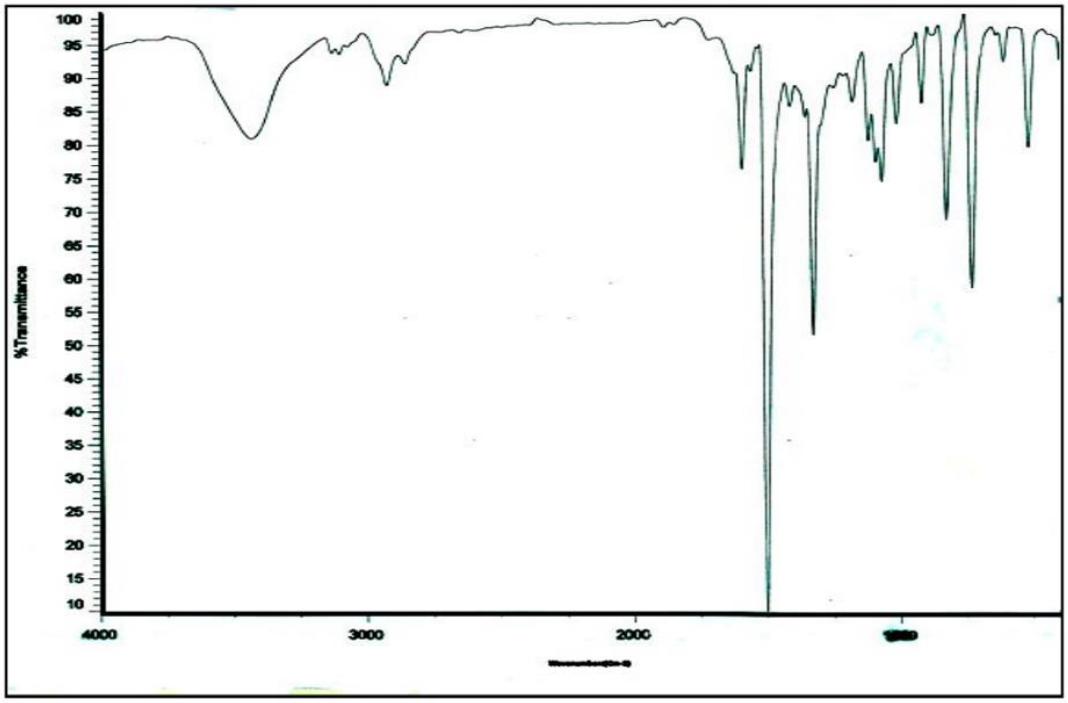

Figure S10. FT-IR of 1-(4-Chlorophenyl)-1H-pyrrole (3e)

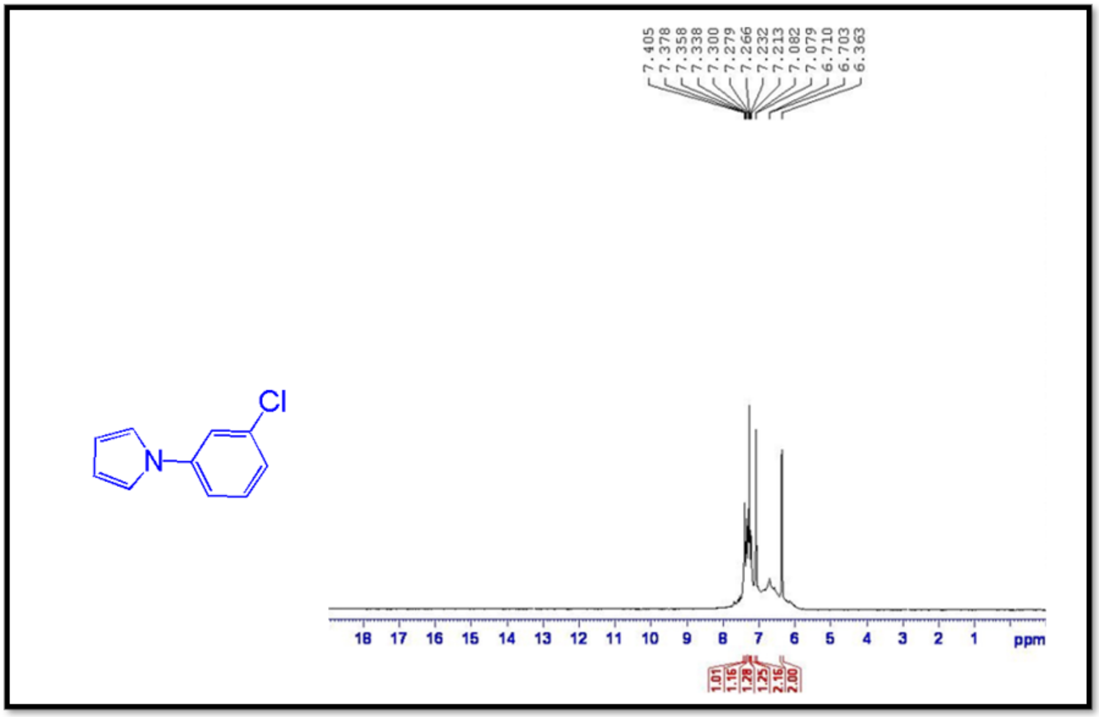

Figure S11. ^{1}H NMR spectra of 1-(3-Chlorophenyl)-1H-pyrrole (3f)

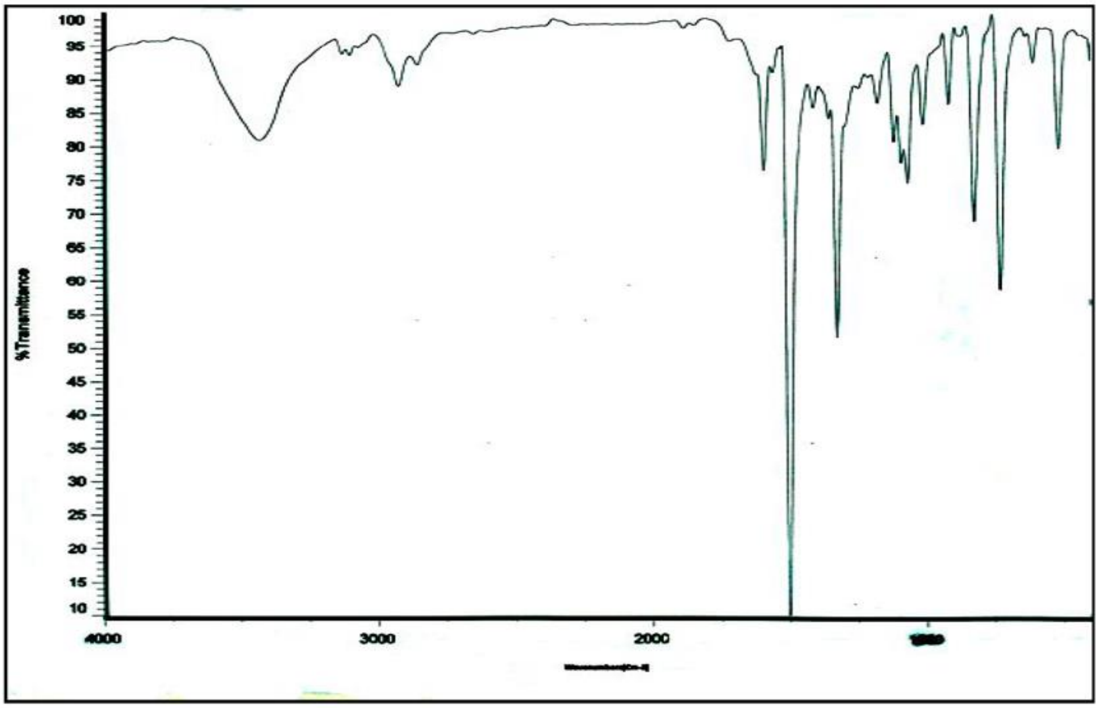

Figure S12. FT-IR of 1-(3-Chlorophenyl)-1H-pyrrole (3f)

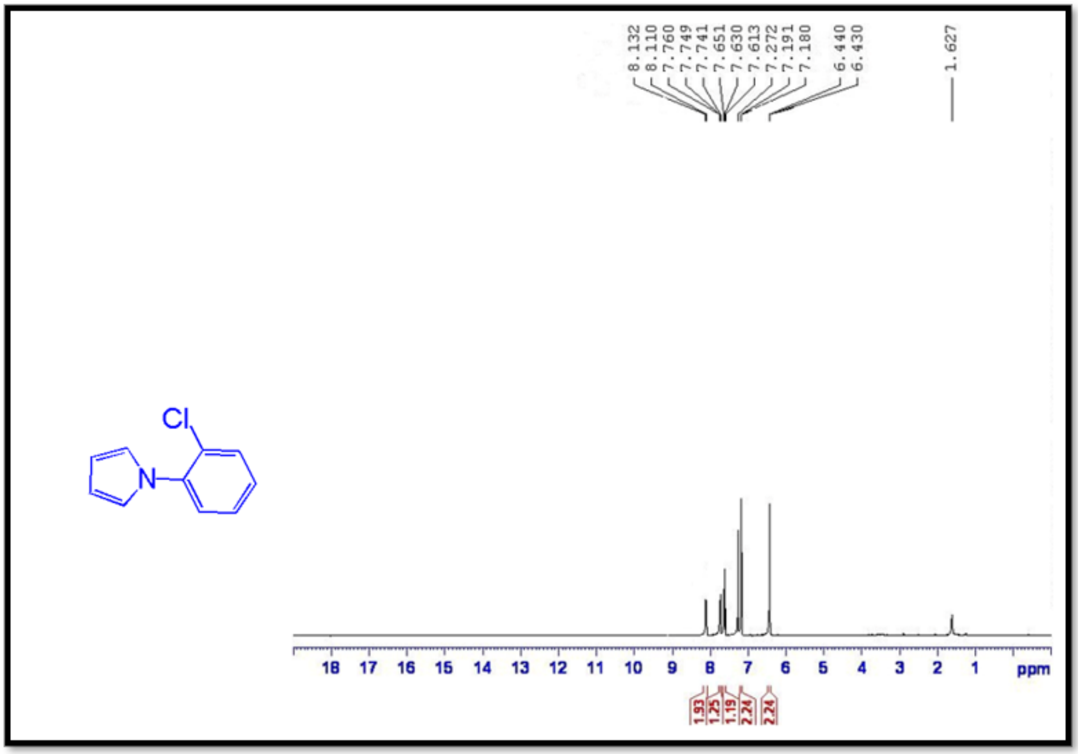

Figure S13. ^{1}H NMR spectra of 1-(2-Chlorophenyl)-1H-pyrrole (3g)

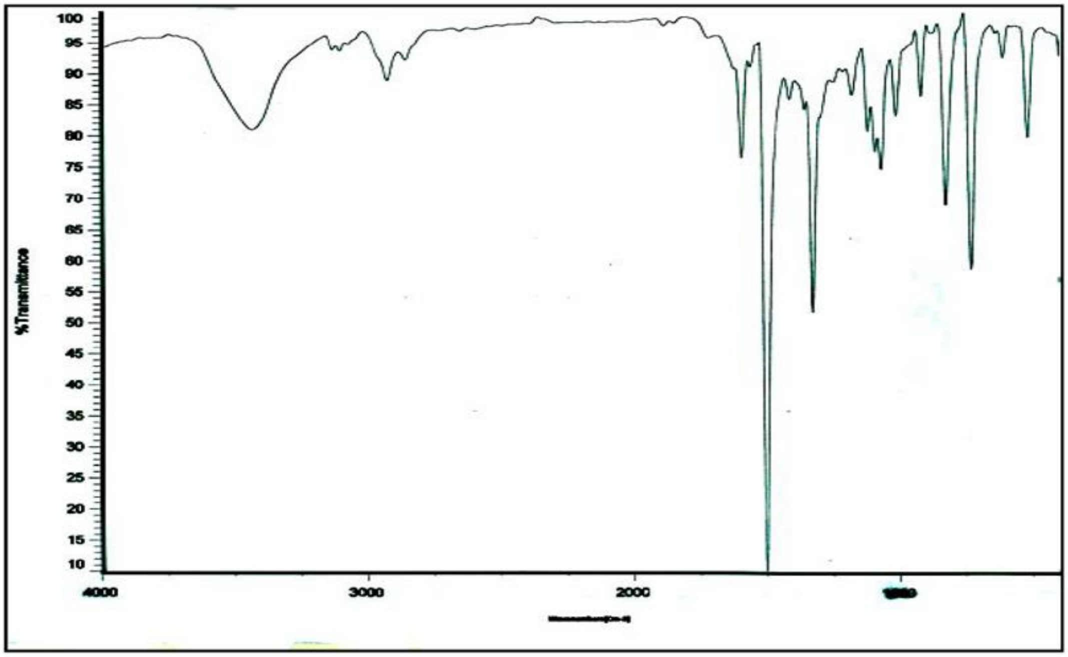

Figure S14. FT-IR of 1-(2-Chlorophenyl)-1H-pyrrole (3g)

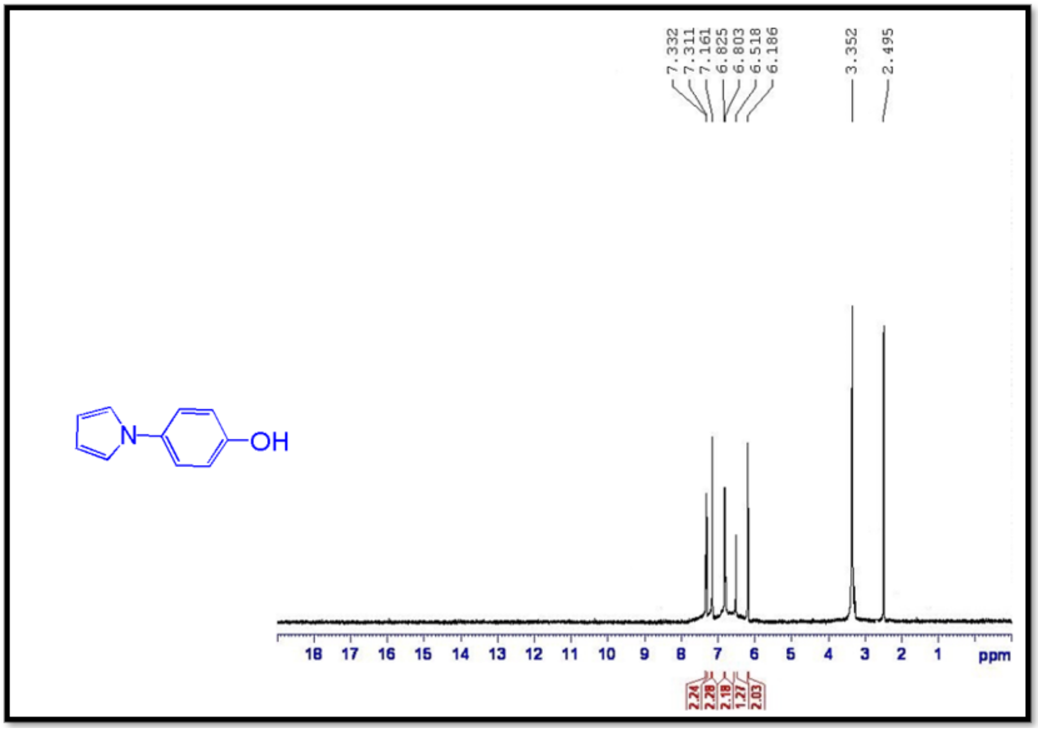

Figure S15. ^{1}H NMR spectra of 4-(1H-pyrrol-1-yl) phenol (3h)

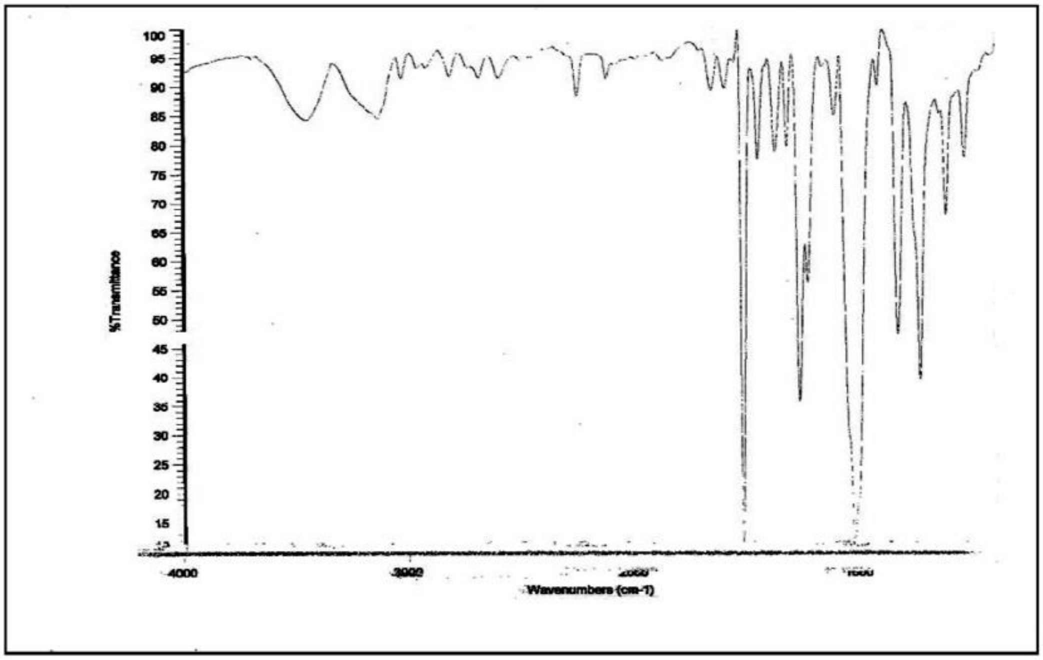

Figure S16. FT-IR of 4-(1H-pyrrol-1-yl) phenol (3h)

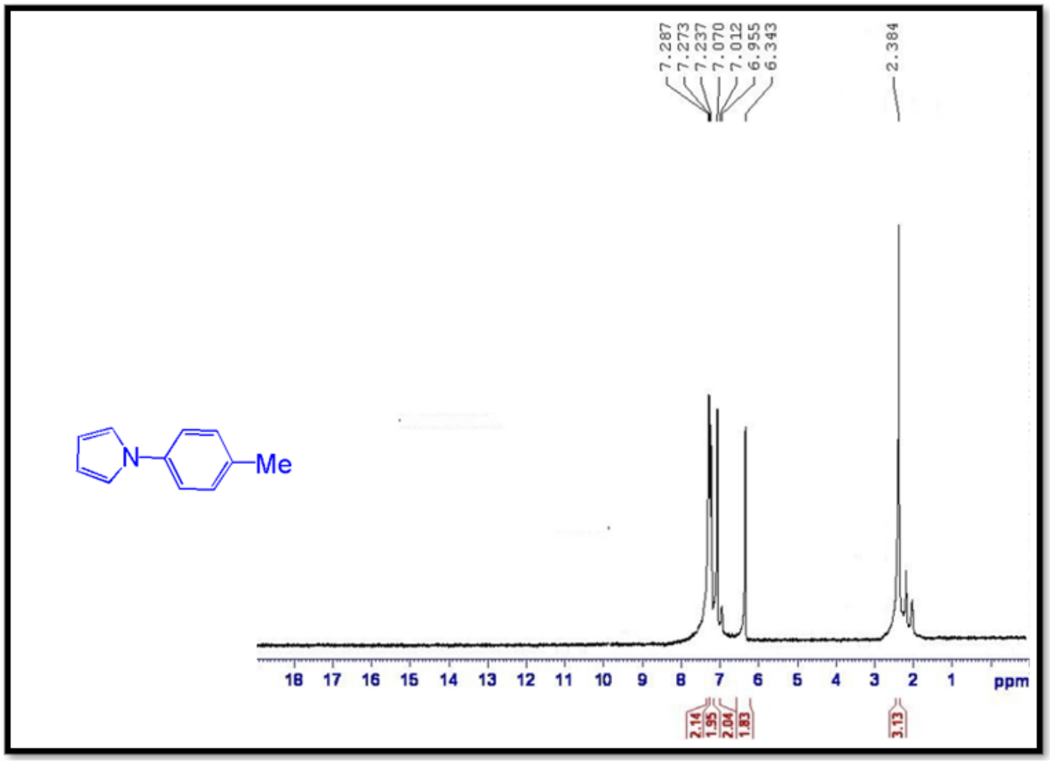

Figure S17. ¹H NMR spectra of 1-(p-tolyl)-1H-pyrrole (3i)

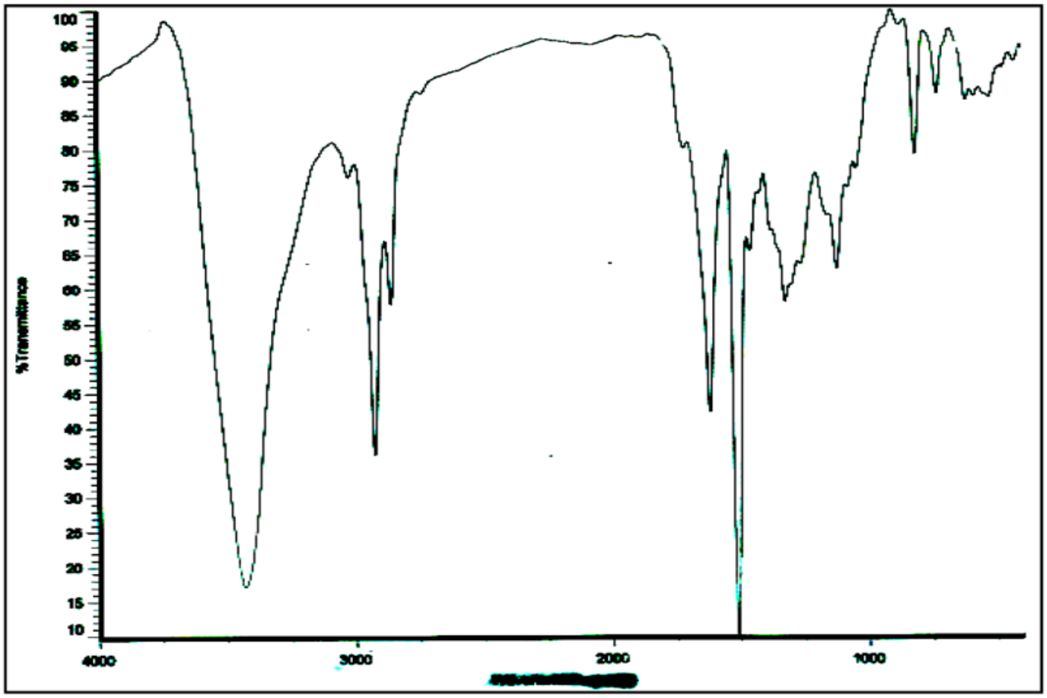

Figure S18. FT-IR of 1-(p-tolyl)-1H-pyrrole (3i)

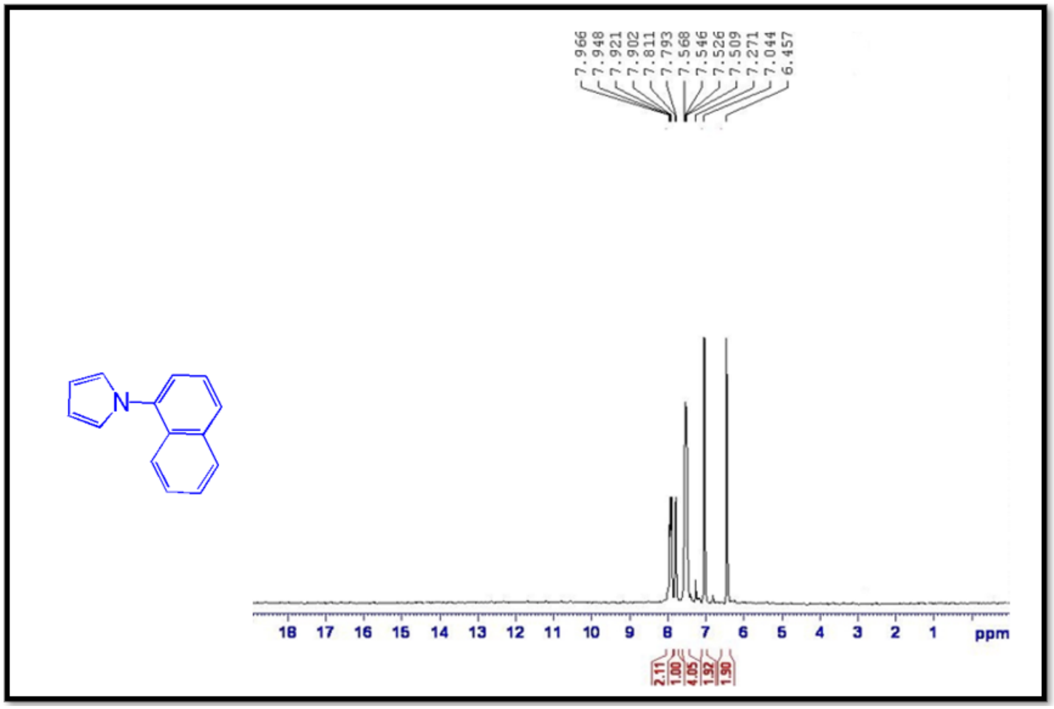

Figure S19. ^{1}H NMR spectra of 1-(naphthalen-1-yl)-1H-pyrrole (3j)

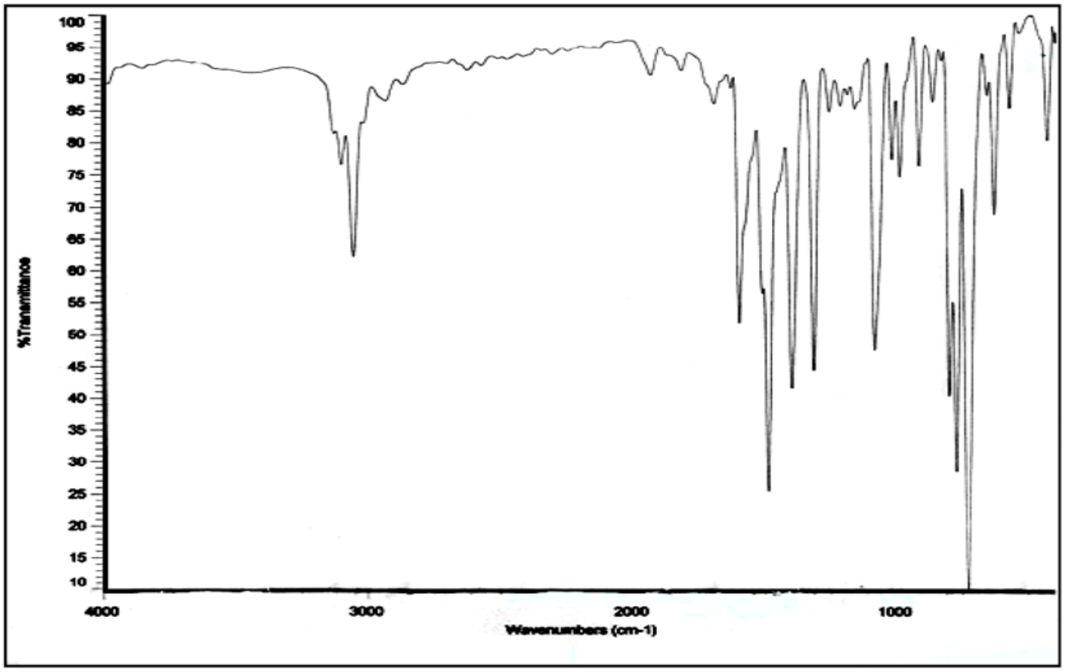

Figure S20. FT-IR of 1-(naphthalen-1-yl)-1H-pyrrole (3j)

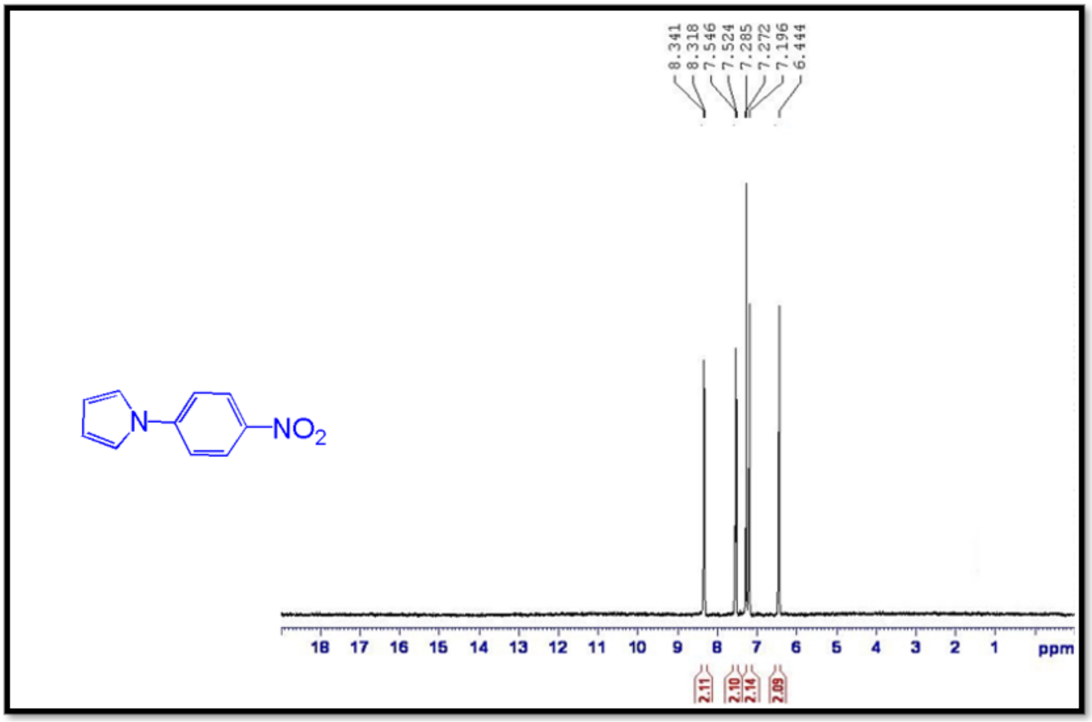

Figure S21. ^{1}H NMR spectra of 1-(4-Nitrophenyl)-1H-pyrrole (3k)

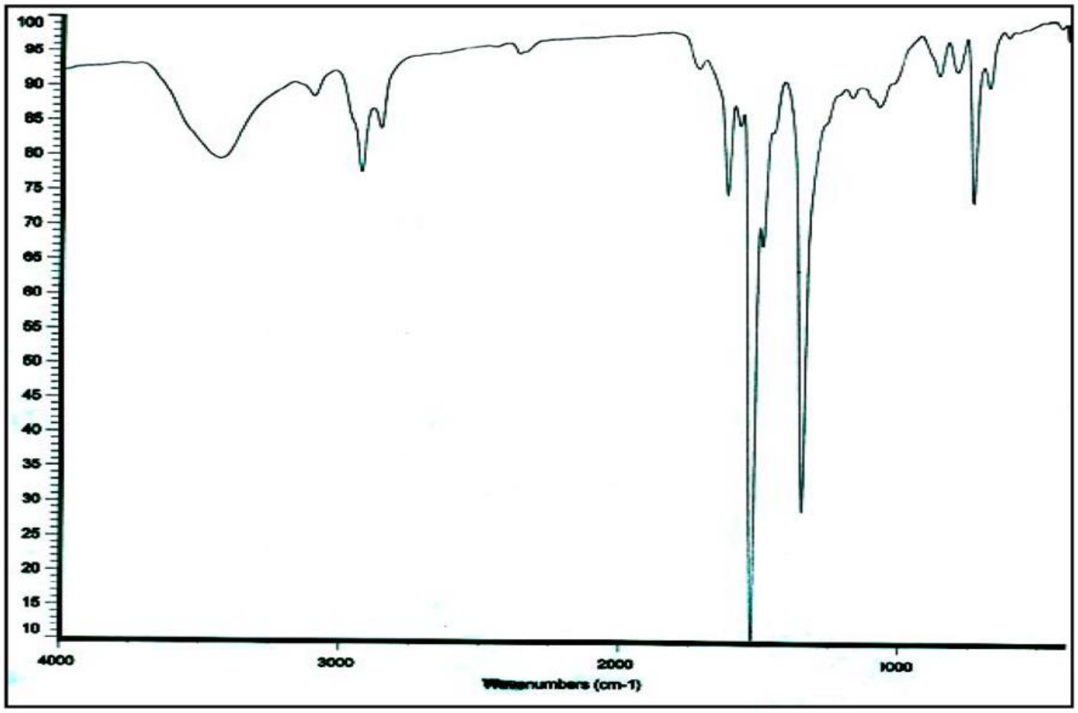

Figure S22. FT-IR of 1-(4-Nitrophenyl)-1H-pyrrole (3k)

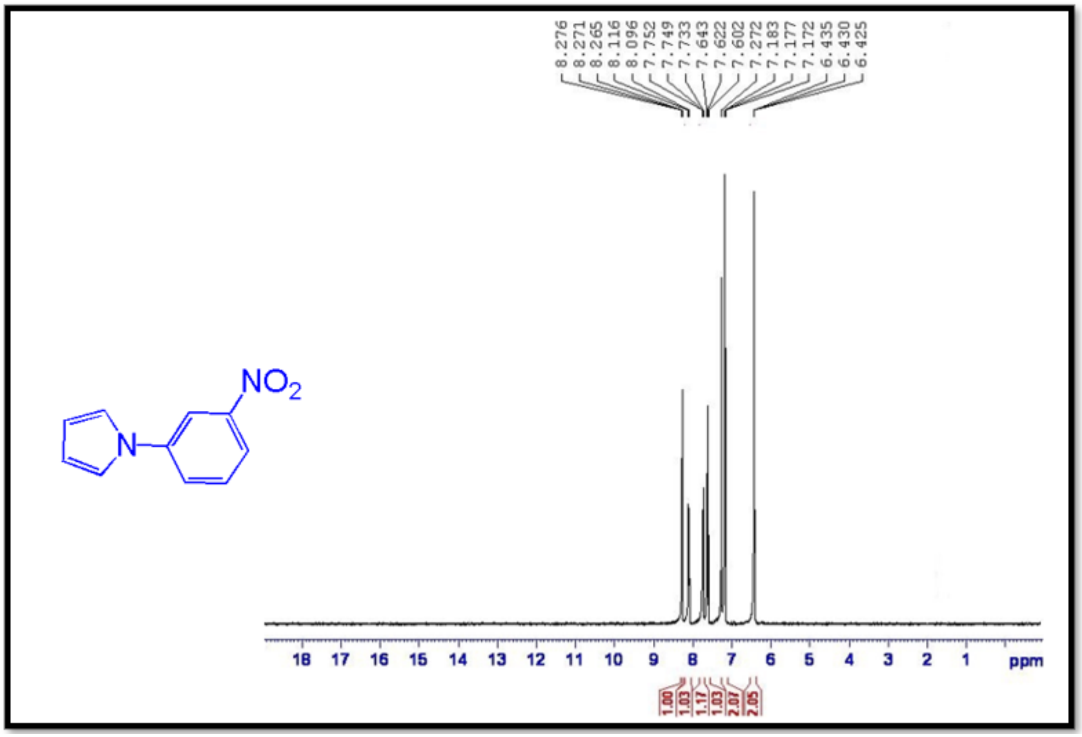

Figure S23. ^{1}H NMR spectra of 1-(3-Nitrophenyl)-1H-pyrrole (3l)

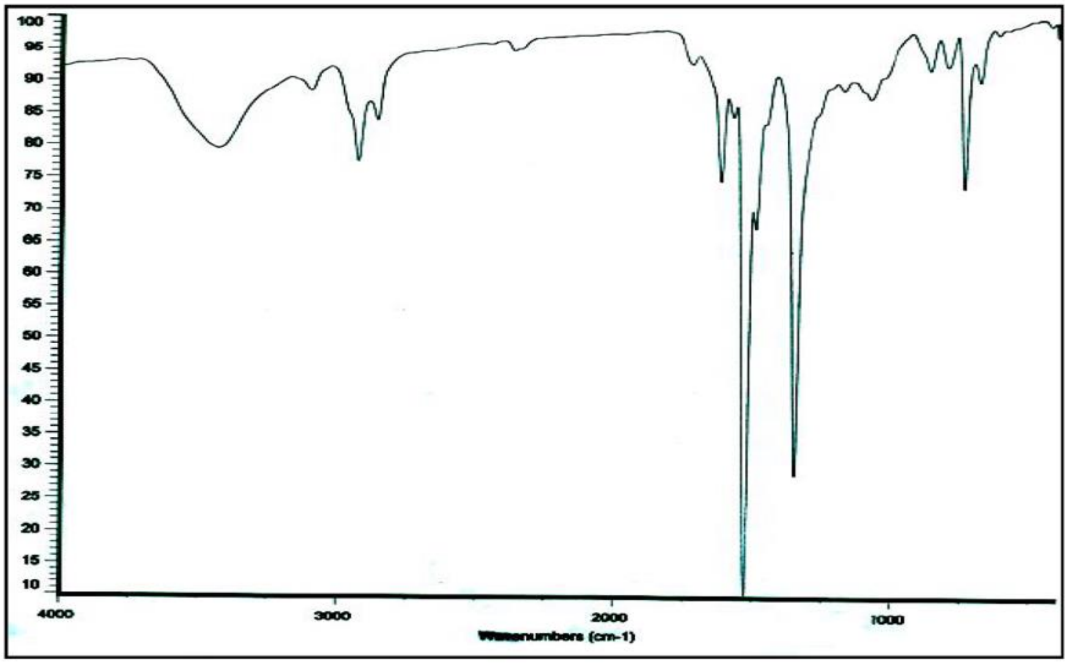

Figure S24. FT-IR of 1-(3-Nitrophenyl)-1H-pyrrole (3l)
